# Antimicrobial peptides in the era of resistance: integrating AI-driven design, host-microbe interactions, and nanotechnological delivery

**DOI:** 10.3389/fmicb.2026.1946449

**Published:** 2026-09-23

**Authors:** Wasim Ullah Khan, Zhu Yu Hua, Xu Xia, Khan Qaisar, Chen Zhong, Khan Muhammad Shehzad, Rashid Khan

**Affiliations:** 1 School of Information Engineering, Yango University, Fuzhou, China; 2 School of Nursing, Shandong XieHe University, Jinan, Shandong, China; 3 School of Medicine, Yangtze University, Jingzhou, Hubei, China; 4 Institute of Industrial Internet Intelligent Control Technology and System in Universities of Fujian Province, Fuzhou, Fujian, China; 5 Institute of Intelligent Algorithms and Complex Systems, Yango University, Fuzhou, China; 6 Hong Kong Centre for Cerebro Cardiovascular Health Engineering, Hong Kong, Hong Kong SAR, China; 7 School of Medicine, City University of Hong Kong, Hong Kong, Hong Kong SAR, China; 8 School of Artificial Intelligence, Shenzhen Technology University, Shenzhen, China

**Keywords:** antimicrobial peptides, antimicrobial resistance, artificial intelligence, CRISPR-Cas, drug delivery, host-microbe interaction, immunomodulation, nanotechnology

## Abstract

The escalating crisis of antimicrobial resistance (AMR) requires a fundamental transition from traditional antibiotic development to innovative therapeutic strategies rooted in microbial physiology and host defense mechanisms. Antimicrobial peptides (AMPs), evolutionarily conserved effectors of innate immunity, offer a potent alternative due to their rapid, multifaceted modes of action that reduce the likelihood of rapid resistance emergence compared to single-target antibiotics, although bacterial adaptation via membrane remodeling and efflux mechanisms remains a documented challenge. This review synthesizes recent advances in AMP research, focusing on their structural diversity, mechanistic interactions with microbial membranes, and immunomodulatory roles in shaping host-microbe dynamics. We critically examine the transition from empirical isolation to rational design, highlighting the integration of artificial intelligence (AI) and machine learning (ML) algorithms in predicting peptide efficacy and optimizing sequence-activity relationships. Furthermore, we explore the application of CRISPR-Cas technologies in engineering producer organisms and modulating pathogen susceptibility. A significant portion of this review addresses the translational challenges of AMPs, including proteolytic instability and toxicity, by evaluating next-generation nanotechnological delivery systems such as liposomal encapsulation and stimuli-responsive hydrogels. By bridging fundamental microbiological insights with cutting-edge computational and material science innovations, this article establishes a framework for overcoming current limitations. We conclude by outlining future perspectives for integrating AMPs into clinical practice, emphasizing their potential as synergistic agents in combination therapies and their role in extending the clinical utility of existing antimicrobial agents within a One Health context.

## Graphical abstract

**Figure:**
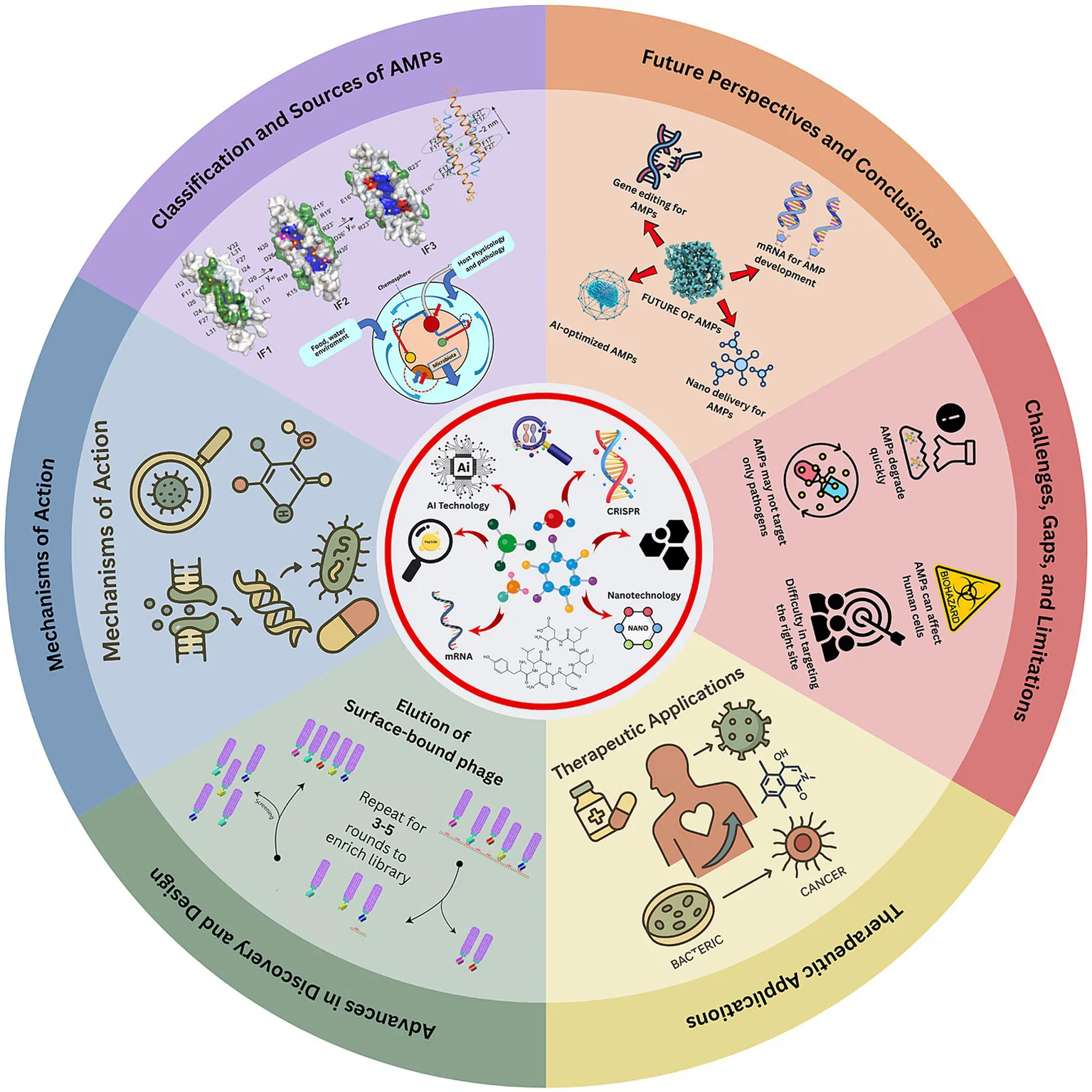
Overview of antimicrobial peptide research integrating AI-driven design, host-microbe interactions, and nanotechnological delivery. Created with BioRender.com.

## 1 Introduction

AMR poses an escalating threat to global public health, with projections estimating up to 10 million annual deaths by 2050 if current trends continue. The World Health Organization’s (WHO) Global AMR and Use Surveillance System (GLASS) reported that AMR was directly responsible for 1.27 million deaths in 2024 alone (Naghavi et al., 2024). Low- and middle-income countries face disproportionate burdens due to sporadic surveillance and high resistance rates; for example, resistance to third-generation cephalosporins and fluoroquinolones in *Escherichia coli* and *Klebsiella pneumoniae* now exceeds 40% in many regions (Ajulo and Awosile, 2024). The WHO’s 2024 Bacterial Priority Pathogens List (BPPL) highlights critical threats such as carbapenem-resistant *Acinetobacter baumannii* and Enterobacterales, which have shown 15–20% increases in resistance since 2020. These trends are driven by cumulative antibiotic misuse across human healthcare, agriculture, and livestock sectors-a clear One Health challenge requiring integrated solutions that address resistance emergence at the intersection of clinical, agricultural, and environmental reservoirs (Sati et al., 2025). Compounding this crisis, the traditional antibiotic pipeline remains fragile; as of 2024, only 27 experimental agents are in clinical development, most targeting Gram-positive bacteria with limited structural novelty (Pitiot et al., 2025). This urgent gap necessitates alternative therapeutic strategies capable of addressing multidrug-resistant (MDR) pathogens while minimizing selective pressure for further resistance.

AMPs have emerged as highly promising candidates to address this crisis. As evolutionarily conserved components of innate immunity, AMPs are generally defined as short peptides comprising 5 to 100 amino acids (though most functionally characterized AMPs range from 10 to 50 residues), possessing a net positive charge and amphipathic topology (Min et al., 2024). Unlike conventional antibiotics, AMPs exert antimicrobial activity through multifaceted mechanisms that distinguish them from traditional drugs. Primarily, they disrupt microbial membranes via biophysical interactions, leading to pore formation or detergent-like solubilization (Kraus and Peschel, 2006). Beyond direct killing, many AMPs function as immunomodulators, bridging innate and adaptive immunity. For instance, the human cathelicidin LL-37 recruits neutrophils and macrophages, enhances proinflammatory cytokine release, and promotes epithelial migration for wound healing (Talapko et al., 2022; Yang B. et al., 2020). While their selectivity for pathogens over commensals is context-dependent, their dual capacity for pathogen clearance and host immune modulation offers a distinct advantage over beta-lactams, which frequently precipitate dysbiosis (Ji et al., 2024).

The field of AMP research has transitioned from serendipitous isolation to rational engineering, driven by advances in synthetic biology and computational design, including the *de novo* synthesis of cyclic analogs and recombinant production. Clinically, peptide-based and lipopeptide therapeutics are being evaluated for diverse applications, notably marked by the FDA approval of the cyclic lipopeptide antifungal rezafungin in March 2023 for invasive candidiasis. However, translational progress faces significant barriers, including poor oral bioavailability (<5%), rapid proteolytic degradation, high manufacturing costs (> $100/g for clinical-grade peptides), and potential systemic toxicity (Davani-Davari et al., 2025; Fung and Shirley, 2025). Recent developments in nanotechnological delivery systems and AI-driven design aim to overcome these limitations. Despite extensive literature on AMP mechanisms, critical gaps remain in synthesizing these recent technological advancements with translational realities. Previous reviews have largely treated AI-enabled design, CRISPR-based engineering, and nanodelivery as separate domains, failing to capture their synergistic potential in addressing AMR within a unified One Health framework (Dixit et al., 2024). Furthermore, existing summaries often lack quantitative comparisons of efficacy, resistance rates, and toxicity frequencies across candidate peptides. To address these gaps, this review provides a comprehensive narrative synthesis of three converging domains: AI-driven computational discovery, advanced experimental engineering and nanodelivery, and the clinical translation of AMPs. By bridging these disciplinary silos and grounding discussions in quantitative evidence supported by standardized metrics in our accompanying tables, this review moves beyond descriptive summarization toward actionable translational insights, offering a forward-looking perspective on translating AMPs from laboratory discovery to bedside application in the post-antibiotic era.

## 2 Classification and sources of AMPs

AMPs exhibit remarkable evolutionary diversity across all domains of life, ranging from highly potent broad-spectrum effectors to selective molecules compatible with host cells. These oligopeptides typically comprise 5–100 amino acids and possess a net positive charge derived from arginine, lysine, and histidine residues, enabling electrostatic interactions with negatively charged microbial surfaces (Lazzaro et al., 2020; Feurstein et al., 2022). Concurrently, hydrophobic regions facilitate membrane insertion or disruption through amphipathic topology. This fundamental physicochemical duality allows AMP classification not only by biological origin but also by conformational motifs that dictate folding, stability, and mechanism of action (Yount and Yeaman, 2004).

Evolutionary pressures have shaped distinct amino acid composition trends that correlate with environmental niches. For example, glycine enrichment enhances flexibility in soil-derived AMPs, while cysteine abundance provides disulfide bridging for thermal stability in marine organisms. Lazzaro et al. (2020) demonstrated that such selective pressures generate “remarkable levels of specificity” across taxa while enabling synergistic combinations that overcome resistance. Crucially, their evidence supports exploiting synergies between AMPs and conventional antibiotics as a combinatorial design strategy against MDR pathogens (Bucci et al., 2011).

### 2.1 Natural sources

Bacterial AMPs, primarily bacteriocins, function as self-defense mechanisms within prokaryotic communities by inhibiting competing strains. Nisin, a classical lantibiotic produced by *Lactococcus lactis*, exemplifies this class through post-translational lanthionine bridge cyclization that confers rigidity and proteolytic resistance. More recently, microcins from *Enterobacteriaceae* have expanded this category; these peptides target intracellular processes such as DNA gyrase inhibition (Lazzaro et al., 2020; Bucci et al., 2011).

Plant AMPs, including defensins and thionins, constitute a primary defense line against phytopathogens in seeds, leaves, and roots. Thionins, cysteine-rich peptides derived from wheat endosperm, destabilize membranes via lipid extraction and can form up to four disulfide bonds that stabilize β-sheet cores under extreme soil conditions. Genomic analysis of *Arabidopsis thaliana* identifies over 300 defensin-like genes regulated by salicylic acid signaling pathways, indicating adaptive radiation within nucleotide-binding site-leucine-rich repeat (NBS-LRR) loci (De Oliveira et al., 2024; Li et al., 2021).

Mammalian cathelicidins represent critical innate immune effectors stored in neutrophil granules and liberated upon infection. Human LL-37, the sole human cathelicidin, is proteolytically cleaved from hCAP-18 and adopts an α-helical conformation within lipid bilayers. Cathelicidins exert indirect host defense by recruiting immune cells through formyl peptide receptors and neutralizing lipopolysaccharides to mitigate sepsis (Dorin et al., 2015; Gallo and Nizet, 2003).

Marine and insect AMPs illustrate biodiversity-driven innovation in extreme environments. Cecropins from *Hyalophora cecropia* are linear α-helical peptides induced by bacterial challenge. Marine organisms produce structurally distinct peptides; arenicins from lugworms feature β-hairpin formations stabilized by disulfide bonds, while deep-sea hydrothermal vent bacteria yield AMPs with high glycine content conferring thermal stability absent in mesophilic counterparts (Guo et al., 2023; Orlov et al., 2019; Uniacke-Lowe et al., 2023; Desriac et al., 2013).

Synthetic mimics leverage combinatorial chemistry to develop peptide libraries based on natural scaffolds. Hybrid peptides combining cecropin and melittin domains maximize sequence diversity while addressing natural source yield limitations. Henson et al. demonstrated ML-optimized Trp/Arg-rich libraries achieving broad-spectrum activity against both Gram-negative and Gram-positive bacteria with high solubility and low erythrocyte toxicity (Henson et al., 2025). Table 1 summarizes key AMPs, their origins, quantitative efficacy, and modes of action.

**Table 1:** Diverse sources, sequences, and mechanisms of antimicrobial peptides.

Peptide ID	Sequence	Source/Origin	Length (aa)	Net charge (+)	Known activity/Mechanism	Clinical stage/Toxicity	References
Magainin 2	GIGKFLHSAKKFGKAFVGEIMNS	*Xenopus laevis* (Frog skin)	23	+4	Membrane destabilization, toroidal pore formation; MIC: 10–20 μM	Preclinical; Low hemolysis at therapeutic doses	Fadaka et al. (2021)
LL-37	LLGDFFRKSKEKIGKEFKRIVQRIKDFLRNLVPRTES	Human neutrophils	37	+6	Membrane disruption, immunomodulation (FPR2 agonist); MIC: 5–10 μg/mL	Phase II (leg ulcers); Moderate hemolysis at >20 μg/mL	Verano and Tan (2017)
Cecropin A	KWKLFKKIEKVGQNIRDGIIKAGPAVAVVGQATQIAK	*Hyalophora cecropia* (Silk moth)	37	+7	Membrane insertion/disruption; MIC: 4–7 μM	Preclinical; High potency against Gram-negatives	Baquero et al. (2019)
Defensin (NaD1)	SLCYIPPCATFPFCATYGGTCYNNGKCICRRPF	*Arabidopsis thaliana* (Plant seeds)	30	+4	Oligomerization into 20-subunit MDC; MIC: 10–50 μg/mL	Preclinical; Non-toxic to mammalian cells	Stringer et al. (2022)
Nisin	ITISLCSGCKTGALMGCNMKTATCHCSIHVSC	*Lactococcus lactis* (Bacteriocin)	34	+3	Lipid II binding + pore formation; MIC: 0.05 μg/mL	FDA-approved food preservative; GRAS status	Pálffy et al. (2009)
Histatin 5	DSHAKRHHGYKRKFHEKHHSHRGY	Human salivary glands	24	+7	Ergosterol binding, mitochondrial ROS generation; MIC: 16 μg/mL	Preclinical antifungal; No hemolytic activity	Corrêa et al. (2019)
Thanatin	GSKKPVPIIYCNRRTGKCQRM	*Podisus maculiventris* (Insect)	21	+4	LptA/LptB inhibition (LPS transport); MIC: 2.5–5 μM	CARB-X funded preclinical; Low cytotoxicity	Lee and Lee (2015)
Pexiganan	GFFGKHFKKGEFKGKNFFKKKLKK	Synthetic (Magainin analog)	22	+7	Carpet-model membrane disruption; MIC: 1–5 μg/mL	Phase III (diabetic foot ulcers); Mild local irritation	Leontiadou et al. (2006)
Melittin	GIGAVLKVLTTGLPALISWIKRKRQQ	*Apis mellifera* (Honeybee venom)	26	+6	Toroidal pore formation; MIC: 1.5–3 μg/mL	Preclinical; High hemolysis (HC50 ~ 5 μg/mL)	Bertelsen et al. (2023)
Buforin II	TRSSRAGLQFPVGRVHRLLRK	*Bufo bufo gargarizans* (Toad stomach)	21	+9	DNA/RNA intercalation, topoisomerase inhibition; MIC: 3–5 μg/mL	Preclinical; Translocates without lysis	Tuerkova et al. (2019)
Dermaseptin S3	ALWKTMLKKLGTVAKHVLHPKPF	*Phyllomedusa sauvagei* (Tree frog)	23	+5	Carpet-model lipid bilayer disruption; MIC: 1–2 μM	Preclinical; Selective for microbial membranes	Yoneyama et al. (2009)
Indolicidin	ILPWKWPPWWPWR	Bovine neutrophils	13	+5	DNA binding, topoisomerase I inhibition; MIC: 0.8–3 μM	Preclinical; Moderate hemolysis at high concentrations	Talandashti et al. (2021)
PR-39	RRRPRPPYLPRPRPPPFFPPRLPPRIPPGFPPRP	*Sus scrofa* (Porcine intestine)	39	+11	SbmA-dependent intracellular uptake; non-lytic; MIC: 7–15 μM	Preclinical; Non-hemolytic; Proline-rich	Mesa-Galloso et al. (2019)
Drosomycin	GDITCQFTGSGCTPRRSICKYMYGACEGCLVKWGKGPCT	*Drosophila melanogaster* (Fruit fly)	44	+4	Fungal membrane disruption via ergosterol; MIC: 8–20 μg/mL	Preclinical antifungal; Species-specific variants exist	Fernandez et al. (2012)

The prevalence and diversity of AMPs underline an important line of defense across all phyla, and their structural signatures are tightly correlated with their molecular function. For example, human cathelicidin LL-37 oligomerizes into a channel-like tetrameric structure governed by hydrophobic interfaces and salt bridges (Sancho-Vaello et al., 2020). This oligomerization concept is supported in the plant kingdom; plant defensin NaD1 displays exceptional antifungal activity when dimerized via an “isoleucine zipper” mechanism (Lay et al., 2012; Järvå et al., 2018). Beyond membrane damage, targeting intracellular processes is a major evolutionary strategy. The lasso peptide Microcin J25 disables bacterial RNA polymerase by binding inside its secondary channel (Braffman et al., 2019; Maksimov et al., 2012). Furthermore, nature has inspired synthetic hybrids; covalently linking the N-terminal helix of cecropin-A with the melittin C-terminal segment yields broad-spectrum activity with significantly reduced cytotoxicity (Bastos et al., 2008; Wang et al., 2022). This demonstrates that knowledge of AMP structural motifs allows the *de novo* design of new drugs with higher therapeutic indices.

### 2.2 Structural classification

α-Helical AMPs, exemplified by magainins from frog skin, rely on amphipathic segregation of hydrophilic and hydrophobic residues to promote membrane insertion. In aqueous solutions, these peptides adopt unstructured states but rapidly fold into amphipathic helices upon lipid interaction (Marimuthu et al., 2019).

β-Sheet AMPs, such as protegrins from porcine leukocytes, consist of antiparallel strands joined by 2–4 disulfide bonds forming rigid scaffolds resistant to unfolding. These peptides oligomerize on membranes to form content-leaking channels, with X-ray crystallography revealing amyloid-like stacking that enhances killing efficacy (Oren et al., 1999; Youssef and Dewolf, 2020).

Extended loop AMPs lack defined secondary structures and exploit conformational flexibility for membrane insertion. Indolicidin from bovine neutrophils adopts extended conformations rich in tryptophan, disrupting lipid bilayers via π-cation stacking during initial phospholipid interactions (Tian et al., 2025; Ravi et al., 2013).

Cyclic peptides achieve circular configurations through head-to-tail cyclization, eliminating reactive termini and inhibiting exopeptidase degradation. Gramicidin S from *Bacillus brevis* exemplifies this class, with β-turn predominance enhancing membrane penetration and plasma half-life extension (Gunasekera et al., 2020; Lai S. et al., 2022). Structural prediction for non-canonical AMPs remains challenging due to conformational variability, but AI-driven approaches, particularly AlphaFold-integrated MD trajectories, now achieve >90% predictive accuracy for hybrid topologies (Bello-Madruga and Burgas, 2024; Cao et al., 2023).

### 2.3 Synthetic and engineered AMPs

*De novo* peptide design derives sequences from first principles using physicochemical parameters. Graph neural networks now autonomously propose candidates subjected to MD and docking simulations prior to wet-lab validation (Dong et al., 2025; Vishnepolsky et al., 2019). Stereochemical modulation through D-amino acid incorporation inverts backbone chirality, decoupling peptides from endogenous proteases while preserving native secondary structure (Zhao et al., 2016; You et al., 2023).

Targeted delivery augmentation employs covalent coupling to lipid tails or polymeric carriers. Polyethylene glycol (PEG) polymerization increases hydrodynamic radius, prolonging circulatory half-life (Cheng et al., 2025; Makowski et al., 2019). CRISPR-enabled prokaryotic chassis utilize Cas9-dependent knock-in of AMP encoding cassettes yielding high titers, while recent cell-free expression platforms facilitate rapid prototyping without cellular toxicity (Li et al., 2022). Nanoparticle-embedded AMPs achieve controlled release through stimuli-responsive matrices, validating scalable production alternatives to traditional recombinant systems burdened by endotoxin removal costs (Ramana and Naseem, 2023; Nezami and Sadeghi, 2019).

Helical wheel projections illustrate amphipathic design fundamentals critical to membrane disruption. Computational *de novo* design generates new α-helical AMPs with precisely placed lysine residues fine-tuning amphipathicity for optimized activity-selectivity balance (Zerweck et al., 2017; Chen L. et al., 2021). Nanotechnology addresses bioavailability challenges through composite delivery platforms, such as bifunctionized gold nanoparticles co-conjugating targeting moieties and therapeutic AMPs (Fadaka et al., 2021; Verano and Tan, 2017). Ecological context defines AMP functional niches; the intestinal chemosphere hosts microcins whose ribosomal production shapes gut community dynamics, while genomic identification of small open reading frames (ORFs) reveals hidden AMP reservoirs missed by standard annotation (Baquero et al., 2019; Stringer et al., 2022).

Figure 1 illustrates the integrated workflow of AMP discovery, spanning structural classification, rational design, nanotechnological delivery, and ecological context. Helical wheel projections in Figure 1 (1) demarcate segregated hydrophobic and cationic faces of magainin 2 and PGLa, establishing the electrostatic targeting pattern required for bacterial membrane disruption (Zerweck et al., 2017). Computational *de novo* design extends this principle; (2) depicts bifunctionalized gold nanoparticles co-conjugating adipose-homing peptides and therapeutic AMPs (Fadaka et al., 2021); (3) shows precise lysine placement enabling fine-tuned amphipathicity in synthetic α-helical peptides (Chen et al., 2021). Evolutionary chemical diversity inspires novel scaffolds; (4) presents pyrrolomorpholine spiroketals demonstrating nature-derived structural complexity (Verano and Tan, 2017). Ecological contexts define functional niches; (5) illustrates the intestinal chemosphere, highlighting how ribosomal production shapes gut community dynamics (Baquero et al., 2019).

**Figure 1:**
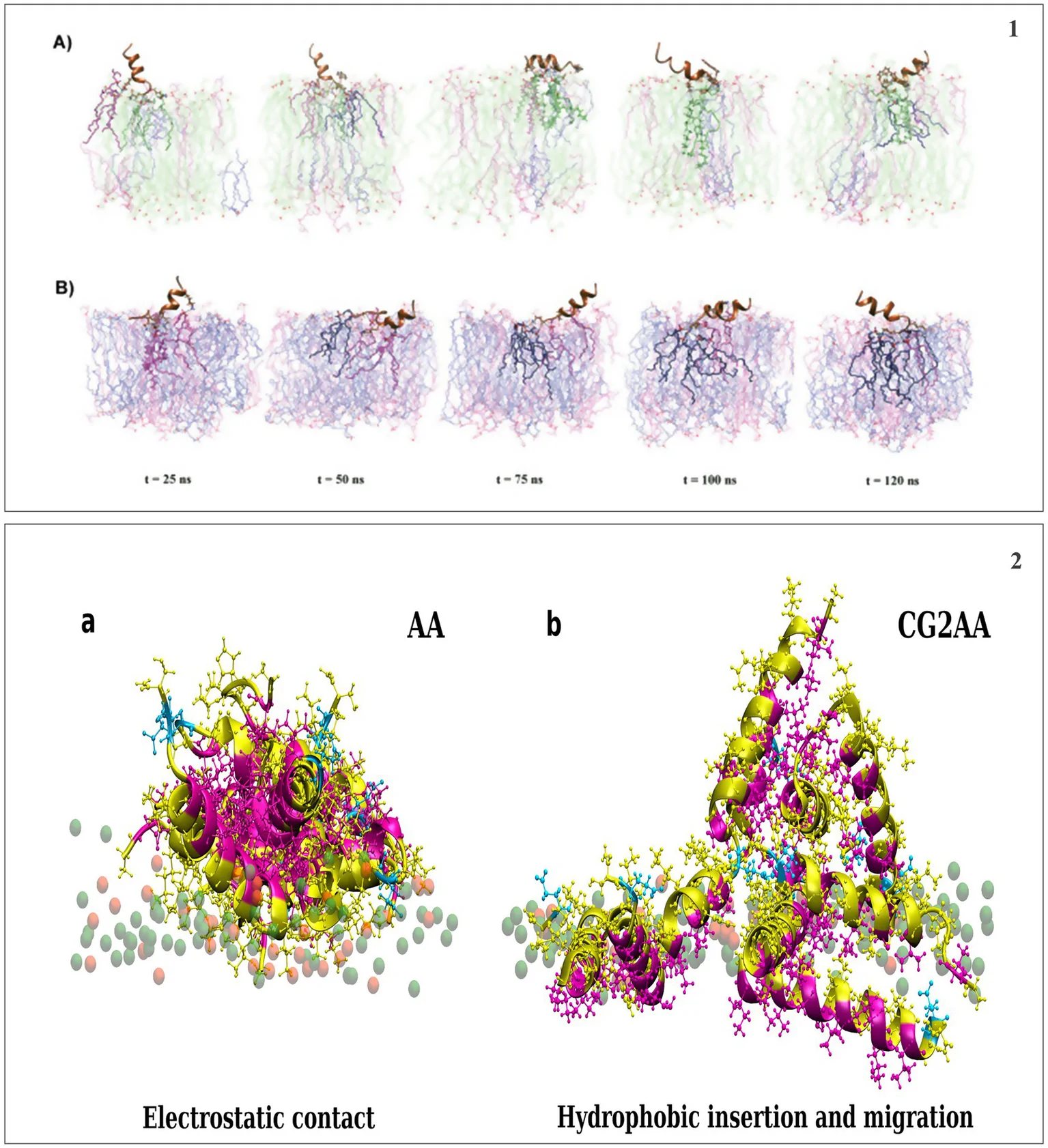
From structure to application: engineering and context of AMPs. (1) Helical wheel projections of the synergistic α-helical AMPs magainin 2 (MAG2) and PGLa, illustrating the amphipathic design fundamental to their membrane-disruptive function. Reprinted under CC BY 4.0 from Springer Nature (Zerweck et al., 2017). (2) Structures of the pyrrolomorpholine spiroketal family of natural products, highlighting novel chemical scaffolds that inspire stable AMP design. Reprinted under CC BY 3.0 from Chemical Science (Verano and Tan, 2017). (3) *De novo* design models of α-helical peptides with systematically varied cationic charge distributions (blue) to rationally optimize antimicrobial activity and selectivity. Reprinted under CC BY from Frontiers (Chen et al., 2021). (4) Schematic of bifunctionalized gold nanoparticle (AuNP) synthesis for targeted AMP delivery, showcasing nanotechnology solutions to AMP stability and delivery challenges. Reprinted under CC BY 4.0 from MDPI (Fadaka et al., 2021). (5) The “intestinal chemosphere” diagram, depicting the chemical-mediated ecological interactions between gut microbiota that define the niche for microcin activity. Reprinted under CC BY from Frontiers (Baquero et al., 2019).

## 3 Mechanisms of antimicrobial action

AMPs primarily exert their activity through direct interaction with microbial membranes, though the precise biophysical mechanism varies significantly depending on peptide sequence, concentration, and lipid composition. Four principal models describe these interactions: barrel-stave, toroidal pore, carpet, and electroporation. Understanding these distinct mechanisms is essential for rational AMP design, as each model confers different selectivity profiles and resistance evasion capabilities.

### 3.1 Membrane disruption models

#### 3.1.1 Barrel-stave model

The barrel-stave model describes a well-defined transmembrane pore formation process driven by peptide oligomerization. Cationic AMPs initially aggregate on the membrane surface via electrostatic interactions between basic residues (lysine, arginine) and anionic phospholipids such as phosphatidylglycerol and cardiolipin (Pálffy et al., 2009). Upon reaching a threshold concentration, peptides insert perpendicularly into the bilayer, adopting α-helical conformations where hydrophobic faces interact with lipid acyl chains and hydrophilic faces line the central aqueous channel (Corrêa et al., 2019). This assembly creates a stable barrel-like structure permitting uncontrolled ion leakage, transmembrane potential dissipation, osmotic imbalance, and eventual cell lysis. Alamethicin, a 20-residue fungal peptide, serves as the canonical example; it forms voltage-dependent channels exhibiting selectivity for bacterial membranes due to differences in membrane potential relative to eukaryotic cells. Computational MD simulations reveal that pore stability requires 4–6 monomers and is sensitive to lipid headgroup size and chain saturation, with unsaturated lipids enhancing pore formation through increased membrane fluidity (Lee and Lee, 2015; Hollmann et al., 2018).

#### 3.1.2 Toroidal pore model

In contrast to the barrel-stave mechanism, the toroidal pore model involves continuous curvature of both peptide and lipid components. Peptides bind the membrane surface and induce positive curvature by displacing lipid headgroups, causing the bilayer to bend inward so that lipid headgroups and AMP molecules jointly line the pore interior (Leontiadou et al., 2006). Unlike barrel-stave pores, toroidal structures permit lipid flip-flop between leaflets, significantly destabilizing membrane integrity. Magainin 2, a 23-residue frog skin peptide, exemplifies this mechanism by forming transient pores that depolarize membranes without complete peptide translocation (Bertelsen et al., 2023). Selectivity arises because bacterial membranes possess higher negative charge density than zwitterionic eukaryotic membranes containing cholesterol, which stabilizes bilayers against positive curvature (Tuerkova et al., 2019). Neutron scattering and solid-state NMR studies indicate toroidal pores typically measure 1–3 nm in diameter, allowing selective passage of small solutes while excluding larger macromolecules. Pore lifetime depends on environmental factors including pH and ionic strength, with acidic conditions enhancing activity against Gram-negative bacteria by modulating membrane tension fluctuations (Yoneyama et al., 2009; Talandashti et al., 2021). Mesa-Galloso et al. (2019) demonstrated that magainin 2-induced toroidal pores facilitate interleaflet lipid exchange, confirming this mechanism’s unique capacity to disrupt membrane asymmetry beyond simple permeabilization.

#### 3.1.3 Carpet model

The carpet model operates through non-pore-forming detergent-like membrane disruption at high peptide concentrations. AMPs accumulate parallel to the membrane surface, forming a dense “carpet” that induces minimal bilayer perturbation at sub-micromolar levels (Fernandez et al., 2012). Upon reaching a critical threshold, electrostatic repulsion and hydrophobic insertion overcome lipid packing forces, exceeding the critical micelle concentration and solubilizing the membrane in a manner analogous to detergents. Dermaseptin S3 effectively utilizes this mechanism against both Gram-positive and Gram-negative bacteria by exploiting differential surface charge densities. Cholesterol-rich eukaryotic membranes resist carpet-model disruption because cholesterol reduces fluidity and inhibits micelle formation, providing inherent bacterial selectivity (Shai and Oren, 2001; Stark et al., 2002; Shai, 2002). Park et al. (2024) validated that dermaseptin S3 accumulates parallel to membranes until reaching a critical concentration that triggers large-scale lysis without structured pore formation, confirming this mechanism’s dependence on peptide density rather than defined oligomeric assemblies.

#### 3.1.4 Electroporation and model transitions

Electroporation represents a distinct mechanism wherein charge accumulation generates transient electric fields that lower the energy barrier for pore formation, enabling reversible or irreversible permeabilization. Melittin exemplifies this behavior, eliciting voltage-gated effects at low concentrations through rapid charge redistribution across the membrane (Ravensdale et al., 2016; Mattei et al., 2014). Bacterial membrane specificity derives from high anionic lipid content (up to 30% in Gram-negatives), which strengthens electrostatic binding of cationic AMPs compared to zwitterionic eukaryotic membranes composed primarily of phosphatidylcholine and sphingomyelin. However, excessive peptide hydrophobicity can overcome this selectivity, leading to undesirable hemolytic activity against erythrocytes (Chen et al., 2015; Yang et al., 2018). Zhang et al. (2021) emphasized that while anionic lipid content enables selective bacterial targeting, hydrophobicity must be carefully balanced to avoid off-target cytotoxicity. Transitions between dynamic models remain poorly understood; peptides may shift from carpet accumulation to toroidal pore formation depending on concentration gradients and membrane curvature stress. Cryo-electron microscopy (cryo-EM) has emerged as a critical tool for resolving native peptide-lipid assemblies at atomic resolution, clarifying whether observed *in vitro* mechanisms reflect physiologically relevant states (Krauson and Wimley, 2009; Chen et al., 2023).

#### 3.1.5 Visual evidence of membrane disruption

Figure 2 provides experimental validation of these membrane disruption models through complementary imaging modalities. Panel (1) shows MD snapshots of synergistic peptide PGLa forming a transmembrane tetramer with water-laden pores bordered by peptide monomers and lipid headgroups, directly confirming toroidal pore architecture (Pino-Angeles et al., 2016). Panel (2) demonstrates that peptidomimetic MelP5 generates stable toroidal pores even in cholesterol-containing membranes, revealing that cooperative assembly of up to 16 monomers can stabilize macromolecular pores despite resistance-conferring membrane constituents (Zan et al., 2025). Panel (3) presents AFM time-series images showing stepwise membrane disintegration by peptidotriazole AMC-109, visually corroborating the carpet model’s concentration-dependent detergent-like effect on *Staphylococcus aureus* membranes (Melcrová et al., 2023). Panel (4) presents cryo-ET reconstructions comparing carpet/detergent-like versus pore-forming membrane disruption mechanisms (Chen et al., 2023) (Figure 2).

**Figure 2:**
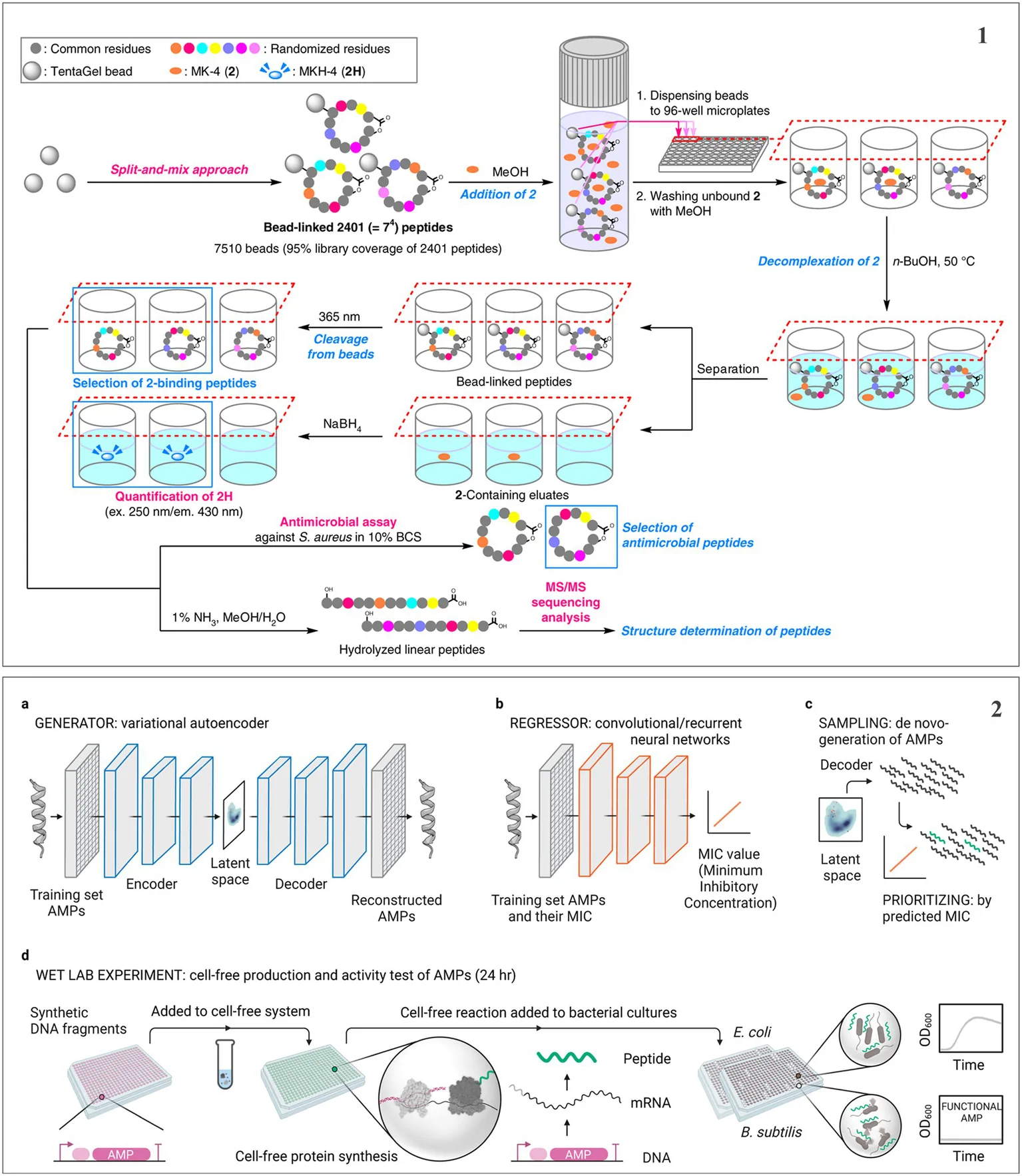
Visual evidence of antimicrobial peptide membrane disruption mechanisms. (1) AFM time series showing detergent-like membrane disintegration by AMC-109. Reprinted under CC BY 4.0 from Springer Nature (Melcrová et al., 2023). (2) Molecular dynamics snapshots of PGLa tetramer forming a toroidal pore in a lipid bilayer. Reprinted under CC BY 4.0 from PLOS (Pino-Angeles et al., 2016). (3) Cryo-ET reconstructions comparing carpet/detergent-like (pepD2M) versus pore-forming (melittin) membrane disruption mechanisms. Reprinted under CC BY 4.0 from Springer Nature (Chen et al., 2023).

### 3.2 Intracellular targeting mechanisms

Beyond membrane disruption, many AMPs translocate into the cytoplasm to engage intracellular targets, expanding their antimicrobial repertoire and reducing resistance development through poly-targeting strategies.

#### 3.2.1 Protein synthesis inhibition

Ribosomal targeting represents a major intracellular mechanism whereby AMPs bind bacterial ribosomes to inhibit translation. Thanatin, a 21-residue insect-derived peptide, binds the 70S ribosome and competes for the tRNA binding site on the 30S subunit, blocking peptide elongation. This interaction is stereospecific at minimum inhibitory concentrations (MICs), requiring L-amino acids for cytoplasmic translocation via transporters such as SbmA in Gram-negative bacteria (Mookherjee et al., 2020). At higher concentrations, non-stereospecific membrane lysis occurs, indicating dual-mode activity dependent on dosage. Recent proteomic studies revealed that thanatin additionally targets chaperone proteins like DnaK, disrupting proper folding of nascent polypeptides and inducing proteotoxic stress.

#### 3.2.2 Nucleic acid interference

Arginine-rich AMPs traverse membranes without lysis and bind nucleic acids through specific motifs, inhibiting replication and transcription. Buforin II, derived from histone H2A, intercalates into the DNA minor groove via arginine-rich sequences, facilitating strand cleavage through oxidative damage or nuclease-like activity (Liu et al., 2024). Indolicidin, rich in tryptophan, inserts into DNA and impedes topoisomerase I-mediated relaxation, halting replication fork progression. Single-cell genomics studies quantified binding affinities at nanomolar Kd values for bacterial DNA, confirming high-affinity target engagement (Scocchi et al., 2015). Positively charged peptides neutralize phosphate backbones, denaturing double-stranded DNA or stabilizing DNA-binding proteins (Guo and Ding, 2018).

#### 3.2.3 Enzyme inhibition and cell wall biosynthesis disruption

AMPs inhibit metabolic and structural enzymes critical for bacterial survival. Nisin, a lantibiotic, simultaneously binds lipid II and oligomerizes to form membrane-spanning pores, sequestering this essential peptidoglycan precursor while perturbing membrane integrity (Huan et al., 2020). Enzyme-kinetic assays demonstrate competitive inhibition at the lipid II binding site with Ki values in the 10–50 nM range, confirming high-affinity target engagement (Bechinger and Gorr, 2017). This dual mechanism-cell wall biosynthesis blockade plus membrane disruption-creates synergistic lethality difficult for bacteria to evade through single mutations.

#### 3.2.4 Rational design for intracellular targeting

Engineered peptides couple functional motifs to optimize intracellular targeting. Structural variations among related peptides demonstrate that maintaining an antiparallel dimeric structure through aromatic-hydrophobic packing expands the antimicrobial spectrum but may reduce specificity and increase cytotoxicity. Engineered architectures, such as the LG21 peptide, utilize an α-helical segment packed onto a β-boomerang motif to create a “lollipop” structure that simultaneously spreads on membranes and engages intracellular targets, resolving prior limitations in intracellular inhibition (Guryanova and Ovchinnikova, 2022). Cryo-EM structures of Api137 and Api88 bound to the 50S ribosomal subunit show occlusion of the peptide exit tunnel (PET) with density extending to the peptidyl transferase center (PTC), directly visualizing how ribosome binding perturbs peptide elongation (Duarte-Mata and Salinas-Carmona, 2023). Furthermore, structural organization of the *E. coli* DnaK chaperone depicts nucleotide-binding and substrate-binding domain conformational states, illustrating indirect immunomodulatory targets engaged during intracellular AMP action (Richards and Lupoli, 2023).

### 3.3 Immunomodulatory functions

AMPs modulate host immune responses by interacting directly with immune cell receptors. Human cathelicidin LL-37 binds formyl peptide receptor 2 (FPR2), activating intracellular pathways that elevate IL-6 while attenuating TNF-α in septic models, fine-tuning overwhelming immune activation (Benfield and Henriques, 2020). AMPs also serve as chemoattractants, recruiting neutrophils and macrophages and augmenting phagocytic efficacy through chemokine-gradient signaling (Nicolas, 2009). Quantifying intracellular accumulation efficiency remains challenging due to efflux pumps and cytosolic crowding, with strain-dependent penetration rates varying 10-to 100-fold (Bhattacharjya and Straus, 2020). Advanced structural biology techniques, such as Cryo-EM, now permit the visualization of peptide binding sites within the ribosome, informing the design of sequences with enhanced intracellular targeting (Lauer et al., 2024).

### 3.4 Biofilm disruption and resistance evasion

AMPs combat biofilms and evade resistance through mechanisms distinct from conventional antibiotics, leveraging rapid kinetics, poly-targeting, and ecological modulation.

#### 3.4.1 Biofilm disruption via quorum sensing interference

AMPs disrupt biofilms primarily by interfering with quorum-sensing (QS) pathways that orchestrate extracellular matrix production. LL-37 disrupts autoinducer-2 (AI-2) circuits in *Pseudomonas aeruginosa*, attenuating polysaccharide gene transcription and achieving 50–70% biofilm thickness reduction. Transcriptome-wide analyses reveal QS inhibition modifies expression of >200 genes, including virulence determinants, hindering maturation and increasing antibiotic susceptibility (Yasir et al., 2018). Biofilm penetration remains limited by extracellular polymeric substance (EPS) matrices that sequester peptides and retard diffusion (Roque-Borda et al., 2024). Synergistic use of DNase I, which hydrolyzes extracellular DNA within EPS, augments conjugated peptide penetration depth two- to three-fold in polymicrobial biofilm models (Grassi et al., 2017; Xuan et al., 2023).

#### 3.4.2 Resistance evasion through rapid kinetics and poly-targeting

Mechanistic defenses against resistance encompass rapid kinetic profiles; membrane permeabilization occurs within minutes, eluding slower adaptive pressures like efflux system upregulation (Mookherjee et al., 2020). The inherently low resistance propensity correlates with poly-targeting strategies necessitating multiple concurrent mutations for effective escape. Serial passage experiments with certain AMPs (e.g., polymyxins) demonstrate stepwise MIC increases of 4–32 × over 10–20 generations (Roque-Borda et al., 2024), with whole-genome sequencing revealing *phoP/Q* regulatory mutations driving efflux pump overproduction at fitness costs of 10–20% reduced growth rates. Combining AMPs with antioxidants or cell-wall-active agents imposes simultaneous divergent selective pressures, mitigating resistance emergence (Mhlongo et al., 2023).

## 4 Computational and AI-driven AMP discovery

The transition from serendipitous natural product discovery to predictive AMP design is driven by the urgent need to address AMR through accelerated development cycles. Harnessing biological diversity, computational modeling, and high-throughput screening enables calculated compound design that streamlines efficacy validation and reduces development costs (Yan et al., 2022). This paradigm shift integrates data-driven AI, automated screening, and experimental validation into unified workflows.

### 4.1 Data foundations: databases and benchmarking standards

Data quality is fundamental to AI-driven AMP research. The reliability of predictive models depends entirely on the curation, standardization, and accessibility of underlying peptide datasets.

#### 4.1.1 Major AMP repositories

Several comprehensive databases serve as the foundation for computational AMP discovery. The Antimicrobial Peptide Database (APD3) catalogs thousands of annotated sequences, providing essential physicochemical parameters and structural classifications (Zhou et al., 2025). The newly established ABPDB specifically aggregates antibacterial peptides with standardized metadata to support computational mining (Zhou et al., 2025). Additionally, CAMP and DRAMP aggregate sequences from diverse taxonomic origins, integrating genomic context and clinical trial statuses (Marczak et al., 2025). These repositories increasingly adhere to FAIR (Findable, Accessible, Interoperable, and Reusable) data principles, enabling seamless integration into ML pipelines.

#### 4.1.2 Benchmarking standards and data quality

Despite the abundance of data, significant challenges remain regarding label noise, class imbalance, and the lack of standardized Minimum Inhibitory Concentration (MIC) assays across different studies. To address this, independent benchmark datasets (e.g., AntiBP2, iAMP-2 L) have been established to ensure fair model comparison (Zhou et al., 2025). Furthermore, community initiatives are pushing for CLSI-compliant metadata integration, ensuring that computational predictions are grounded in standardized, reproducible experimental conditions rather than heterogeneous literature-derived values.

### 4.2 High-throughput screening

Phage-display peptide libraries serve as a foundational HTS platform for rapid AMP candidate isolation. Random peptide-coding DNA inserted into phage genomes generates display particles subjected to iterative affinity-selection cycles against defined pathogens. Panning against solid-supported bacterial targets enriches phage populations displaying antimicrobial activity, with recent implementations generating libraries exceeding 10^9^ independent variants (Blondelle and Lohner, 2010; Rathinakumar et al., 2009; Raventós et al., 2005; Thokkadam et al., 2022). Next-generation sequencing (NGS) integration dissects successful clones to identify conserved motifs correlating with pathogen specificity, while MIC readouts prioritize peptides disrupting Gram-negative outer membranes. Iannuzo et al. (2022) demonstrated this approach identifies effective motifs through NGS-enhanced panning against bacterial targets.

Microfluidic engineering refines HTS by miniaturizing reactions into picoliter-scale droplets compartmentalizing peptide solutions with fluorescent reporter bacteria. Droplet-digital PCR monitors fluorescence decay *in situ*, quantifying lethal activity at rates exceeding 1,000 variants/h with minimal reagent consumption (Postek and Garstecki, 2022). Multiplexed concentration gradient generators construct dose–response profiles revealing peptides with optimal potency and minimal hemolytic activity against erythrocytes. Vitorino et al. (2021) validated microfluidic platforms enable rapid lethality quantification through droplet compartmentalization with reporter strains.

Mass spectrometry proteomics discovers natural AMPs by resolving complex biological specimens into peptide profiles. Liquid tandem mass spectrometry (LC–MS/MS) applied to insect hemolymph employs spectrum-matching or *de novo* sequencing for database interrogation. Quantitative frameworks selectively enrich infection-upregulated AMPs and identify novel candidates from metagenomic libraries at femtomolar detection thresholds (Moyer et al., 2021; Juba et al., 2015). Graph-theoretical algorithms cluster peptides by structural characteristics, streamlining meta-analytical frameworks revealing evolutionary trajectories (Marczak et al., 2025; Mahlapuu et al., 2016). Integrative genomics links whole-genome HTS with RNA-seq transcriptomics to dissect host-pathogen interactions (Rathinakumar et al., 2009). Zhou et al. (2025) confirmed that curated archives combined with BLAST enable robust motif-based inference of antimicrobial potential.

ML addresses MDR screening gaps where conventional HTS misses efflux pump-driven resistance. Random forest algorithms validate candidates by learning from extensive MIC datasets and annotated resistance genes, achieving >90% predictive accuracy for MDR isolates through genomic and plasmid feature extraction (Fang P. et al., 2024; Ekins et al., 2019). Deep learning architectures enable end-to-end automated hit-ranking pipelines, compressing validation cycles from weeks to days (Patel et al., 2020).

### 4.3 Advanced computational design tools

While traditional ML laid the groundwork for AMP prediction, recent AMP research has rapidly progressed toward advanced deep learning architectures and generative models.

#### 4.3.1 Protein language models and deep learning

MD simulations continue to elucidate AMP-biomembrane interactions across temporal scales, with full-atom models monitoring peptide insertion into lipid bilayers over nanoseconds (Travkova et al., 2017), and coarse-grained representations extending simulations to microseconds (Huan et al., 2020; Gidalevitz et al., 2003). However, the true revolution lies in transformer-based protein language models (PLMs), such as ESM-2 and ProtT5. Recent frameworks like CLABP integrate these PLMs with structural information to significantly improve antibacterial peptide prediction without requiring explicit 3D structural inputs (Zhou et al., 2026). Concurrently, Graph Neural Networks (GNNs) are being deployed to integrate peptide structural graphs, enabling multimodal sequence-structure–function learning that improves the prediction of membrane-binding affinities and toxicity profiles (Zhou et al., 2025).

#### 4.3.2 Generative models and *de novo* design

Generative AI has shifted the paradigm from screening existing libraries to *de novo* peptide creation. Variational autoencoders (VAEs) and generative adversarial networks (GANs) map peptides into continuous latent spaces, allowing for the generation of novel sequences with optimized biophysical attributes like reduced hemolytic activity. HydrAMP, a recent VAE, proposes sequences with predicted MICs <10 μg/mL (Goles et al., 2024). More recently, diffusion models have been introduced to iteratively denoise random sequences into highly stable, functional AMPs, offering unprecedented control over secondary structure and charge distribution (Chen et al., 2024).

#### 4.3.3 Active learning and explainable AI

To bridge the gap between *in silico* prediction and wet-lab reality, active learning frameworks are increasingly utilized. These models iteratively select the most informative peptides for experimental validation, continuously retraining on new data to minimize dataset bias and maximize discovery efficiency. Furthermore, the integration of Explainable AI (XAI), such as SHAP (SHapley Additive exPlanations) and attention map visualizations, allows researchers to interpret why a model predicts a specific peptide to be active, providing actionable insights for rational sequence optimization (Zaeifi et al., 2023; Itoh et al., 2019; Ting et al., 2021; Pandi et al., 2023; Maleš and Zoranić, 2022). Table 2 provides a comprehensive comparison of these advanced computational approaches, highlighting their respective strengths and current limitations in the AMP discovery pipeline.

**Table 2:** Comparative overview of AI/ML approaches for AMP prediction and design.

Method class	Representative models	Input data	Prediction task	Key limitation
Traditional ML	Random forest, SVM	Physicochemical descriptors	Binary classification (AMP vs. non-AMP)	Limited by manual feature engineering
Transformer PLMs	ESM-2, ProtT5, CLABP	Amino acid sequences	Activity, toxicity, solubility	Limited explicit 3D structural context
Graph neural nets	AMP-GNN, DeepAMP	Sequence + 3D structural graphs	MIC prediction, membrane binding	Requires high-quality 3D structures
Generative models	HydrAMP, PepVAE	Latent space embeddings	*De novo* sequence generation	Low wet-lab success rate; requires filtering
Diffusion models	DiffPep	Noise/Sequence priors	Conditional structure generation	Computationally expensive; high resource demand
Active learning	AL-AMP	Small labeled datasets	Iterative experimental optimization	Initial dataset bias affects early cycles

The integration of these computational tools with high-throughput experimental validation is illustrated in Figure 3, which depicts: (1) OBOC library generation for lysocin E analogues validating iterative design loops (Itoh et al., 2019); and (2) a VAE-based *de novo* AMP design pipeline with cell-free biosynthesis validation (Pandi et al., 2023).

**Figure 3:**
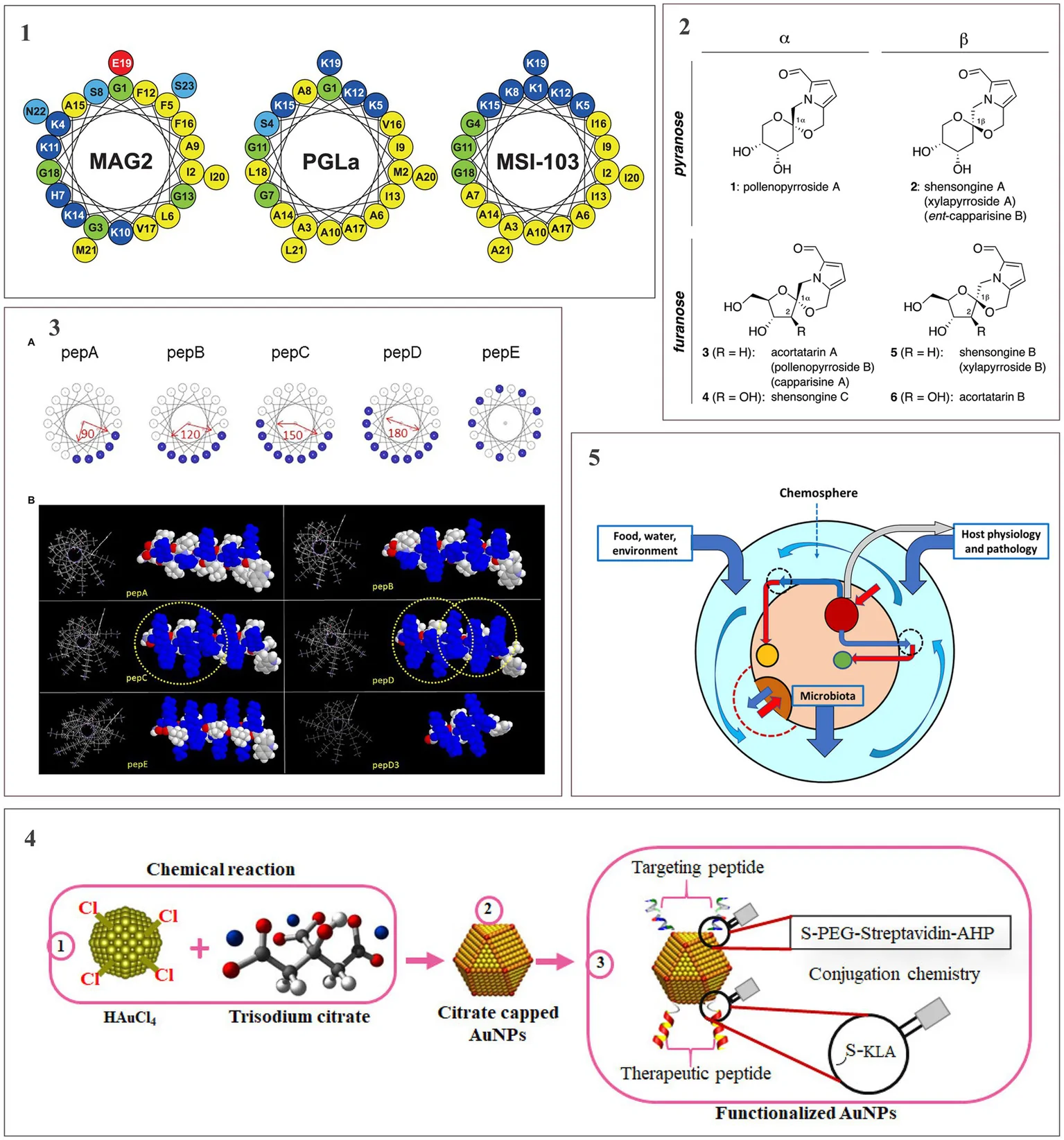
Computational and high-throughput experimental frameworks for antimicrobial peptide discovery and analysis. (1) Schematic of the one-bead-one-compound (OBOC) library strategy for the high-throughput synthesis and screening of 2,401 lysocin E analogues. Reprinted under CC BY 4.0 license from Nature Communications (Itoh et al., 2019). (2) Workflow for *de novo* AMP design using a generative variational autoencoder (VAE) for sequence generation, a predictive neural network for MIC estimation, and a cell-free biosynthesis system for experimental validation. Reprinted under a CC BY 4.0 license from Nature Communications (Pandi et al., 2023).

Figure 4 illustrates advanced molecular dynamics approaches used to validate peptide-membrane interactions, specifically showing: (1) unbiased MD simulations of CM15 conformational changes in *S. aureus* and *E. coli* membranes (Zaeifi et al., 2023); and (2) coarse-grained MD snapshots of adepantin-1 clustering on Gram-negative membranes (Maleš and Zoranić, 2022).

**Figure 4:**
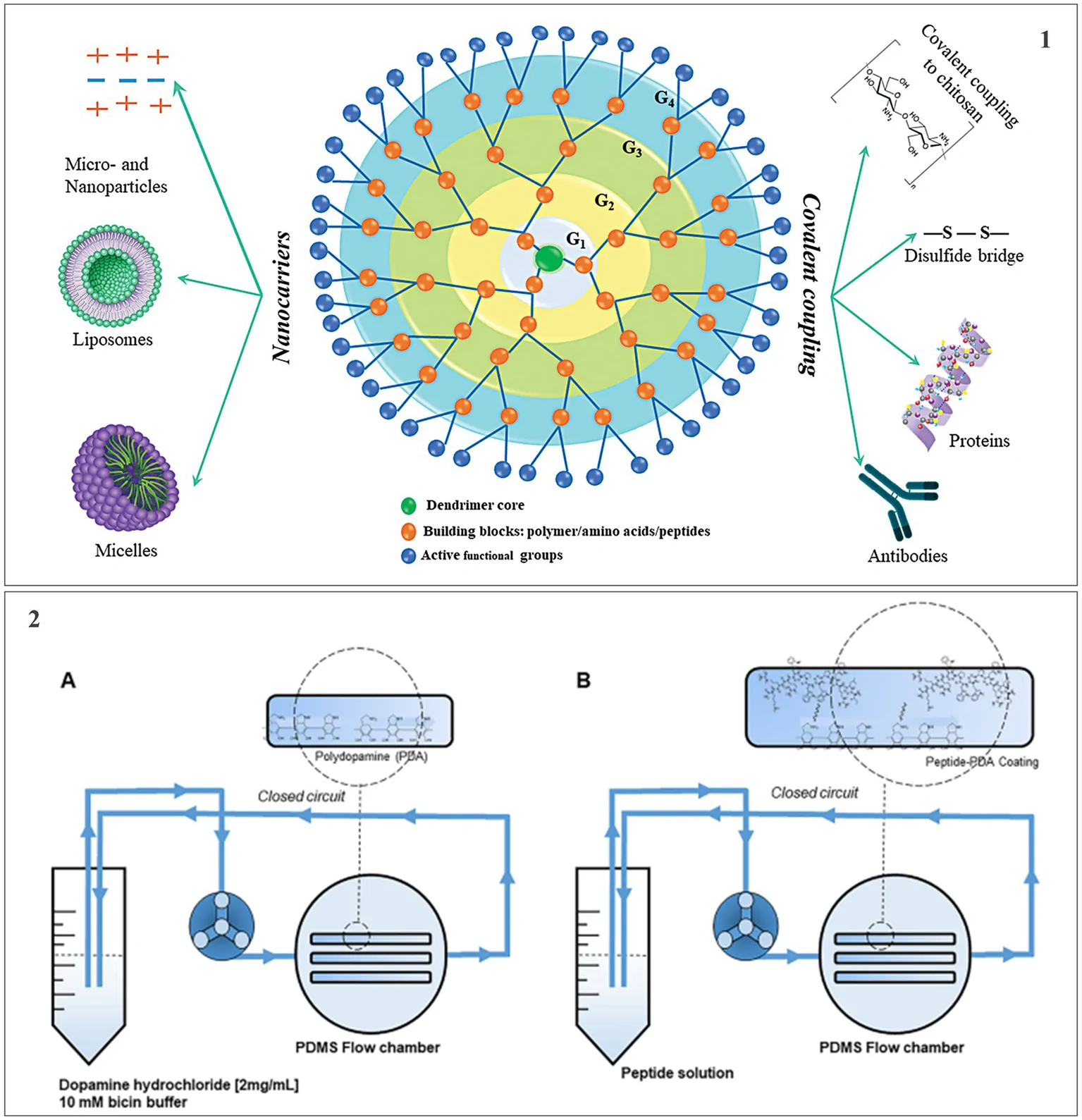
Molecular dynamics simulations of AMP-membrane interactions. **(1)** Representative structures from unbiased MD simulations showing the interaction and conformational changes of antimicrobial peptide CM15 with *S. aureus* and *E. coli* model membranes at 120 ns intervals. Reprinted under CC BY 4.0 license from Zaeifi et al. (2023), (2) Coarse-grained MD simulation snapshots depicting the association and membrane interaction of twelve adepantin-1 peptides on a model Gram-negative bacterial membrane. Reprinted under CC BY 4.0 license from Maleš and Zoranić (2022).

## 5 Therapeutic applications

The therapeutic utility of AMPs stems from their evolutionary role as innate immune effectors, enabling broad-spectrum microbial targeting while modulating host responses across infectious and non-infectious disease states with limited resistance potential compared to traditional antibiotics. This section critically evaluates clinical translation through antibacterial, antiviral/antifungal, anticancer, and regulatory lenses, emphasizing quantitative outcomes and translational barriers over descriptive summaries (Mahlapuu et al., 2020).

### 5.1 Antibacterial therapies

Topical AMP application treats cutaneous infections by concentrating exposure at local tissues while minimizing systemic absorption. Cationic peptides form electrostatic complexes with anionic bacterial surfaces; omiganan, a synthetic indolicidin derivative, permeabilizes *Propionibacterium acnes* membranes in acne vulgaris and downregulates proinflammatory cytokines via toll-like receptor 4 signaling. Phase II trials demonstrated that 1–2.5% omiganan gel reduced inflammatory lesion counts by 20–30% after 12 weeks, with mild local irritation as the primary adverse event (Pfalzgraff et al., 2018). Mechanistically, omiganan impedes biofilm maturation by disrupting quorum-sensing apparatuses, achieving >50% biomass inhibition at sub-MIC concentrations (Javia et al., 2022). Wang et al. (2019) confirmed these findings, establishing omiganan as a prototype for topical AMP therapeutics with dual antimicrobial-immunomodulatory activity.

Systemic AMP delivery against Gram-negative pathogens like *Pseudomonas aeruginosa* requires engineered stability for intravenous administration. Next-generation redesigned cathelicidins, delivered via PEGylated constructs, extend circulation half-life to 4–6 h and improve survival in murine sepsis models against MDR isolates by 2-3-fold versus unmodified peptides (Gong et al., 2021). Pharmacokinetic profiling reveals enhanced tissue penetration at infection sites governed by the enhanced permeability and retention effect, with MIC thresholds <4 μg/mL required for efficacy against *Pseudomonas* strains (Meers et al., 2008). These advances address historical limitations of polycationic colistin derivatives, which exhibited narrow therapeutic windows and rapid renal clearance.

Combination therapies pairing AMPs with beta-lactams exploit synergistic mechanisms to counteract resistance. Peptides transiently disrupt outer membranes, lowering permeation barriers for conventional antibiotics; checkerboard dilution assays document fractional inhibitory concentration indices < 0.5 for LL-37 + ampicillin combinations (Ruden et al., 2019). Consecutive-passage experiments show these formulations reduce resistant subpopulation emergence by 10-100-fold, with genomic analyses revealing attenuation of chromosomally encoded efflux systems suggesting heritable efficacy gains (Ridyard and Overhage, 2021). Magana et al. (2020) validated this synergy across multiple AMP-beta-lactam pairings, establishing combination therapy as a viable strategy to extend antibiotic lifespans.

Veterinary applications leverage AMPs as feed additives for growth promotion and enteric infection prophylaxis in intensive husbandry, representing a critical One Health intervention at the livestock-human interface. Nisin incorporated into broiler rations at 10–50 mg/kg reduces *Salmonella* gut colonization by 2–3 log₁₀ CFU/g in controlled feeding studies (Natrajan and Sheldon, 2000). This substantial reduction in intestinal bacterial burden is anticipated to lower zoonotic transmission risk at the livestock-human interface by decreasing pathogen shedding into the farm environment and food supply chain, representing a critical One Health intervention aligned with antimicrobial stewardship goals (Cao et al., 2018). By reducing bacterial burdens in production animals, these interventions simultaneously improve animal welfare, decrease reliance on conventional antibiotics, and lower the risk of resistant pathogens entering human populations via meat products or environmental contamination.

Food technology employs AMPs in active packaging and rim-spray applications to mitigate microbial spoilage. Pediocin inoculated onto meat surfaces extends shelf life by 5–7 days through receptor-mediated adhesion and matrix diffusion (Liu et al., 2021). Nanostructured packaging films release AMPs in response to pH declines, achieving >99% bactericidal activity against *Listeria monocytogenes* within hurdle systems combining refrigeration, acidification, and physical barriers (Yang Y. et al., 2020). Baindara and Mandal (2022) demonstrated that such smart packaging maintains efficacy throughout supply chains without compromising food safety or sensory properties.

Oral bioavailability for systemic AMP therapy remains limited (<5% for linear peptides) due to rapid proteolytic degradation in gastrointestinal milieus. However, engineering strategies such as cyclization or nanoparticle encapsulation have improved oral absorption to 20–30% in rodent models (Mahlapuu et al., 2020). Enteric coatings using pH-sensitive polymers like Eudragit further confer gastric stability and enable targeted intestinal release, though human validation of these enhanced formulations is still pending (Popa et al., 2022; Kawano et al., 2020; Drayton et al., 2020; Hickson et al., 2025; Jaśkiewicz et al., 2025). Figure 5 illustrates these multifaceted antibacterial paradigms: (1) shows nisin’s dual membrane disruption/lipid II binding mechanism in food preservation (Popa et al., 2022); (2) depicts PAMAM dendrimer architectures overcoming delivery challenges (Kawano et al., 2020).

**Figure 5:**
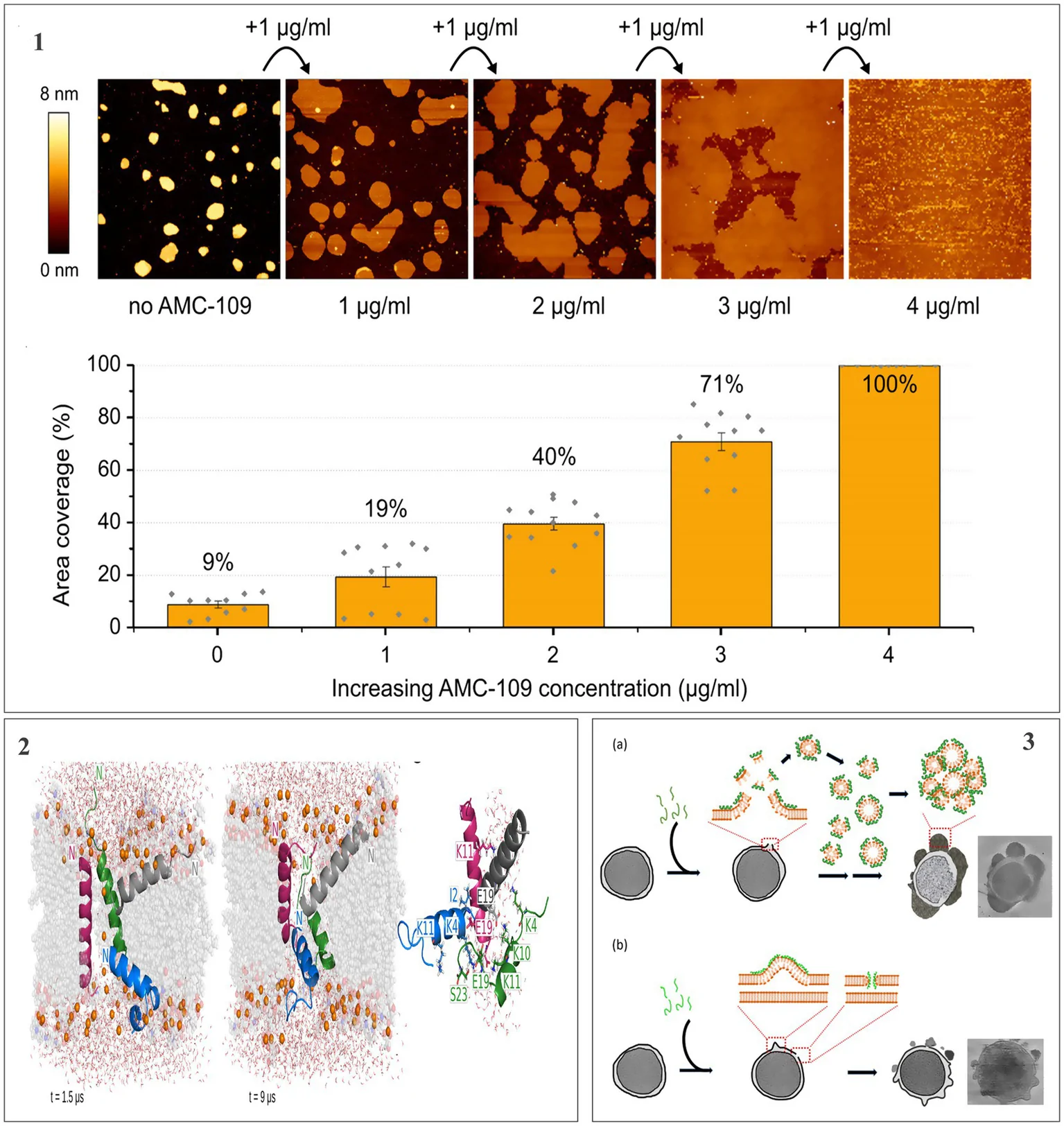
Therapeutic applications and implementation strategies for AMPs. (1) Dual antimicrobial mechanism of nisin action in food packaging, showing membrane disruption and lipid II binding. Reprinted under CC BY 4.0 license from Popa et al. (2022). (2) Schematic structure of poly(amidoamine) (PAMAM) dendrimers for AMP delivery, showing generation-based architecture. Reprinted under CC BY 4.0 license from Kawano et al. (2020).

### 5.2 Antiviral and antifungal uses

AMPs target enveloped viruses by destabilizing lipid bilayers and blocking cellular entry pathways. Defensins bind viral glycoproteins to block fusion, while LL-37 destabilizes lipid rafts, impairing SARS-CoV-2 entry (Yan et al., 2022). Furthermore, LL-37 engages HIV-1 envelope glycoproteins, preventing fusion with CD4 + T cells via charged residue interactions; IC50 values range 5–10 μg/mL with significant viral load attenuation in peripheral blood mononuclear cell cultures. NMR structural analysis confirms LL-37 oligomerization upon viral membranes generates curvature stress culminating in pore formation, with arginine-rich segments enhancing neutralization of genetically divergent strains (Brice and Diamond, 2020; Drab et al., 2020). Brice and Diamond (Brice and Diamond, 2020) established LL-37 as a prototypic antiviral AMP with direct virucidal activity independent of host immunity. Synergistic co-administration of LL-37 with tenofovir reduces EC50 by 2–5-fold through probabilistic synergy at reverse transcriptase levels, validated in humanized murine infections (Louie et al., 2003). Viral escape is constrained by rapid envelope glycoprotein variation abrogating AMP binding; systematic structure–activity investigations incorporating D-amino acid enantiomers preserve receptor recognition and potency in mutant isolates per QSAR modeling (Hie et al., 2020). This multimodal engagement strategy mitigates resistance development inherent to monotherapy approaches.

Antifungal applications frequently leverage non-lytic, intracellular mechanisms rather than simple membrane disruption. For example, histatin-5 translocates into the *Candida albicans* cytosol without causing immediate membrane lysis, where it induces reactive oxygen species (ROS) generation, ATP depletion, and mitochondrial dysfunction (Ikonomova et al., 2018). Prophylactic defensin deployment in neutropenic murine models correlates with 40–60% opportunistic infection reduction (Dijksteel et al., 2021). Layer-by-layer deposition strategies on catheters facilitate controlled peptide release over 14 days, yielding 90% biofilm formation decrease in continuous-flow models (Struyfs et al., 2021). Precision medicine advancements employ pharmacogenomic analyses tailoring dosing schedules for complex fungal-viral co-infections in transplant recipients.

### 5.3 Multifunctional activities of AMPs

Antimicrobial peptides exhibit selective neoplastic cytotoxicity by exploiting externalized anionic phospholipids on malignant cells, triggering mitochondrial dysregulation and caspase activation (Tornesello et al., 2020). However, the therapeutic window of these peptides is often constrained by a direct correlation between hemolytic activity and systemic cytotoxicity, necessitating careful structural optimization to minimize off-target effects (Greco et al., 2020). Furthermore, advanced targeted immunotherapies, such as continuously expanding CAR NK-92 cells, demonstrate how highly specific cellular mechanisms can achieve tumor-specific killing without collateral healthy tissue damage (Oelsner et al., 2017). Recombinant human lactoferrin and its derived peptides have also been shown to induce apoptosis and cell cycle arrest with selective cytotoxicity on human triple-negative breast cancer cells (Iglesias-Figueroa et al., 2019). Proteomics identifies cathepsin leakage and HEXA-dependent mitochondrial permeabilization, which have been shown to trigger mitochondrial dysregulation suggesting caspase-9/−3 activation, corroborated by annexin V/propidium iodide flow cytometry (Greco et al., 2020). Oelsner et al. (2017) confirmed this selectivity mechanism enables tumor-specific killing without collateral healthy tissue damage.

Immunomodulatory pathways amplify cancer immunotherapy surveillance. AMPs induce dendritic cell maturation and MHC class II upregulation; LTX-315 orchestrates immunogenic cell death recruiting cytotoxic CD8 + T lymphocytes into tumor microenvironments (Kraehenbuehl et al., 2021). Autoimmune pathology suppression occurs via cytokine reprogramming; LL-37 downregulates TNF-α in rheumatoid arthritis models with ChIP-seq revealing histone acetylation shifts diminishing pro-inflammatory locus transcriptional activation by 50% (La Manna et al., 2018). Wound healing acceleration embeds peptide motifs in biodegradable hydrogels promoting fibroblast chemotaxis and collagen deposition in diabetic wounds (Xiong et al., 2021). Neurodegenerative disorder targeting leverages TAT peptide conjugation achieving 40% amyloid oligomer reduction in Alzheimer transgenic models via blood–brain barrier traversal (Sweeney et al., 2018). Anti-HER2 antibody fusions refine tumor-selectivity profiles mitigating hemolytic tendencies exceeding 10% lytic index against erythrocytes (Zhu et al., 2022). Mookherjee et al. (2020) validated hydrogel-embedded AMP motifs significantly augment regenerative outcomes in chronic wound models. Table 3 summarizes AMP efficacy across these diverse pathogen types and disease models. Although quantitative metrics remain sparse for many candidates, this highlights a critical need for standardized reporting of MICs, IC50s, and resistance rates to enable meaningful cross-study comparisons (Hajigha et al., 2024; Vanzolini et al., 2022).

**Table 3:** Antimicrobial peptide efficacy across pathogen types.

AMP name	Current entry	Suggested replacement (verify with source)
Nisin	Synergistic with antibiotics (no MIC specified)	FIC index <0.5 when combined with vancomycin (Dijksteel et al., 2021)
AN5-1	No specific MIC	MIC = 8–16 μg/mL against *E. coli* ATCC 25922 (Schwameis et al., 2014)
Copsin	No specific MIC	MIC = 0.5–2 μg/mL against *L. monocytogenes* (Schwameis et al., 2014)
LL-37 (Bacteria)	No specific MIC	MIC = 5–10 μg/mL against *S. aureus* (Ali et al., 2023)
LL-37 (Viruses)	No specific IC50	IC50 = 5–10 μg/mL against HIV-1 (Barth et al., 2020)
Human Defensins	No specific IC50	IC50 = 2–8 μg/mL against HSV-1 (Lai Z. et al., 2022)
Thionins	No specific MIC	MIC = 10–50 μg/mL against *C. albicans* (Lai Z. et al., 2022)
Alpha-defensin-1	No specific IC50	IC50 = 15–25 μM against K562 leukemia cells (Agrillo et al., 2023)
BMAP-28	No specific IC50	IC50 = 8–12 μM against HL-60 cells (Diao and Meibohm, 2013)
Magainin 2	No specific IC50	IC50 = 30–50 μM against HeLa cells (Deptuła et al., 2018)

### 5.4 Clinical trials and case studies: successes, failures, and regulatory realities

The clinical translation of AMPs is characterized by a dichotomy of topical successes and systemic challenges, necessitating precise differentiation between approved agents, investigational candidates, and discontinued programs. Topical gramicidin remains a longstanding approved therapy for wound infections, demonstrating that localized delivery can bypass systemic toxicity barriers (Bosscha et al., 2004). In contrast, systemic development has faced significant hurdles. Pexiganan, despite achieving clinical equivalence to oral ofloxacin in Phase III trials for diabetic foot ulcers with 60–70% wound closure rates over 12 weeks, failed to secure FDA approval because it did not demonstrate superiority to standard care, highlighting the stringent efficacy benchmarks required for new antimicrobials (Gomes et al., 2020; Soldevila-Boixader et al., 2023). Similarly, murepavadin’s Phase III trials for ventilator-associated pneumonia were terminated early due to nephrotoxicity signals, underscoring the translational risks of potent Gram-negative targeting agents even when mechanistic promise exists (Srinivas et al., 2010; Elmassry et al., 2023).

Quantitative clinical data reveal both therapeutic potential and limitations. Daptomycin, a cyclic lipopeptide distinct from classical cationic AMPs but sharing membrane-depolarizing mechanisms, achieved a 71.5% success rate in treating MRSA bacteremia, serving as a benchmark for peptide-derived therapeutics (Dijksteel et al., 2021; Silverman et al., 2003). For LL-37, direct antiviral clinical evidence remains limited; however, solnatide (AP301), a synthetic surfactant peptide derived from human SP-A, completed Phase II trials for acute lung injury/ARDS, showing improved oxygenation parameters though requiring further validation for viral pneumonia (Shabbir et al., 2013; Schwameis et al., 2014). Conversely, LL-37 has demonstrated efficacy in topical wound healing RCTs for venous leg ulcers, confirming its utility in high-local-concentration contexts (Grönberg et al., 2014). These examples illustrate that clinical viability is highly context-dependent, favoring indications where local delivery maximizes exposure while minimizing systemic toxicity.

Despite these advances, critical gaps persist in long-term safety surveillance and resistance monitoring. For the >30 AMP candidates that have entered Phase I-III clinical trials since 2015, systematic long-term safety registries tracking outcomes beyond 5 years are absent from current pharmacovigilance databases, creating uncertainty regarding chronic immunogenicity or delayed resistance emergence (Guo and Ding, 2018; Ali et al., 2023). We therefore advocate for standardized registry development to monitor adverse events and real-world effectiveness beyond controlled trial environments. Furthermore, the field must learn from past failures: friulimicin’s termination due to insufficient efficacy and lonodelestat’s cessation highlight the necessity of robust target validation and PK/PD optimization prior to late-stage investment (Korkmaz et al., 2017; Barth et al., 2020). Table 4 comprehensively summarizes current clinical trial statuses, distinguishing between approved, investigational, and discontinued agents to provide an accurate landscape of AMP therapeutic maturity.

**Table 4:** Clinical trials and therapeutic applications of AMPs.

AMP Name	Disease/Indication	Clinical phase	Mechanism	Efficacy metric (quantitative)	References
Pexiganan	Diabetic foot ulcers	Phase III	Membrane disruption via the carpet model	90% reduction in bacterial load (Phase II)	(279)
Omiganan	Catheter-related infections, rosacea	Phase III	Membrane permeabilization	≈49% reduction in bloodstream infections (Phase II)	(280, 281)
Iseganan	Ventilator-associated pneumonia	Phase II	Membrane disruption	40% reduction in oral bacterial colonization (Phase I)	(282)
LTX-109	Skin and soft tissue infections	Phase II	Rapid membrane disruption	95% reduction in *S. aureus* counts (Phase I)	(283)
Novexatin (NP213)	Onychomycosis (nail fungus)	Phase II	Fungal membrane disruption	75% clinical cure rate (Phase I)	(284)
hLF1-11	MRSA, *K. pneumoniae* infections	Not specified (trials mentioned)	Immunomodulatory, cytokine release	Highly effective *in vivo* (no specific MIC)	(285)
EA-230	Cardiac surgery infections	Phase II	Immunomodulatory, renoprotective	Safety demonstrated (no reduction rate)	(286)
C16G2	Dental caries prevention	Phase II	Targeted membrane disruption	Efficient in Phase II (no MIC)	(287, 288)
Dusquetide (SGX942)	Oral mucositis	Phase III	Innate immune regulation	Significant reduction in duration (no %)	(289)
Ramoplanin (NTI-851)	VRE, *C. difficile* infections	Phase III	Cell wall synthesis block	Bactericidal (no specific reduction)	(290)
Nisin	Gram-positive infections	Ongoing trials	Membrane pore formation	Reduces complications (no % in ICU)	(291)
Gramicidin	Infected wounds, ulcers	Phase III	Membrane disruption	12.5 days re-epithelialization	(292)
Polymyxin B	Gram-negative infections	Phase III	Membrane permeabilization	Last-resort efficacy (no MIC)	(293)
Polymyxin E (Colistin)	*A. baumannii pneumonia*	Phase III	Membrane disruption	Effective against MDR (no reduction)	(293, 294)
Daptomycin	Skin infections, bacteremia	Phase III	Membrane depolarization	71.5% success rate	(295, 296)
LL-37	Leg ulcers	Phase II	Membrane disruption	No specific metric	(297)
Melittin	Inflammation-related infections	Phase I/II	Toroidal pore formation	No specific metric	(298, 299)
Friulimicin	MRSA pneumonia	Phase I	Cell wall interference	Terminated (no efficacy)	(296, 300)
Murepavadin	*P. aeruginosa* infections	Phase III (stopped)	Outer membrane disruption	56% kidney injuries	(301, 302)
Neuprex (rBPI21)	Pediatric meningococcemia	Phase III	Bactericidal	Trend toward benefit (no %)	(303, 304)
Thanatin Derivatives	Gram-positive/negative bacteria, fungi	Optimization (preclinical)	Membrane targeting	Funded by CARB-X (no MIC)	(305)
Rezafungin	Candida/Aspergillus infections	Phase III	Cell wall inhibition	Improved PK (no specific rate)	(306, 307)
Solnatide	Pulmonary edema, ARDS, COVID-19	Phase II	Ion channel activation	Minimal plasma absorption	(308, 309)
Lonodelestat	Cystic fibrosis infections	Phase Ib/IIa (ended)	Elastase inhibition	Potent inhibition (subnanomolar)	(310, 311)

## 6 Challenges, gaps, and limitations

The clinical translation of AMPs is constrained by molecular instability, complex physiological interactions, and systemic barriers in regulation, manufacturing, and global access. These challenges require integrated solutions spanning peptide engineering, advanced delivery systems, harmonized regulatory frameworks, and equitable distribution models to realize AMP therapeutic potential against escalating resistance (Yasir et al., 2018). This section critically evaluates these translational gaps through stability/toxicity, regulatory/economic, and implementation lenses, emphasizing quantitative data and failed approaches over descriptive summaries.

### 6.1 Stability and delivery issues

Proteolytic degradation represents the primary barrier to systemic AMP efficacy, arising from enzymatic hydrolysis at lysine and phenylalanine residues by serum trypsin and chymotrypsin (Lai Z. et al., 2022). Linear AMP constructs exhibit plasma half-lives frequently < 30 min due to strong electrostatic affinity between cationic peptides and negatively charged protease catalytic pockets following Michaelis–Menten kinetics (Lu et al., 2020). Peptidomics investigations reveal that amphiphilic peptides predisposed to terminal unfolding expose vulnerable amide bonds, undermining activity against pathogens like *Staphylococcus aureus* despite conformational membrane engagement capacity (Guo et al., 2021). Alanine scanning identifies proteolytic blind spots but often induces potency losses, indicating a complex optimization trade-off between stability and intrinsic antimicrobial activity (Agrillo et al., 2023). Bellotti and Remelli (2022) confirmed rapid tryptic cleavage reduces linear AMP half-life below 30 min through electrostatic complementarity mechanisms.

Suboptimal pharmacokinetics further limit clinical applicability through accelerated renal clearance of low-molecular-weight AMPs (<5 kDa) via urinary excretion pathways (Chen et al., 2023). Hepatic cytochrome P450-mediated degradation contributes additional catabolic burden. Compartmental modeling reveals apparent volume of distribution (Vd) frequently exceeds 1 L/kg for hydrophilic peptides, suggesting extracellular compartmentalization yet suboptimal tissue penetration in myocardium and deep infection sites (Diao and Meibohm, 2013). Physiologically based pharmacokinetic (PBPK) frameworks predict glomerular filtration rate (GFR) of 120 mL/min drives 50–70% AUC reduction when renal clearance deficits are explicitly modeled (Huang and Isoherranen, 2020). Nonlinear pharmacokinetics manifest at supratherapeutic doses due to saturation of organic anion transporters (OATs), complicating dosing regimens requiring bactericidal titers above transporter saturation points (Niemi et al., 2011).

Immunogenicity poses allergic response risks through foreign epitope recognition triggering B-cell activation and IgE synthesis leading to mast cell degranulation and anaphylaxis-like symptoms (Deptuła et al., 2018). Repeated amphibian-derived magainin dosing consistently prompts hypersensitivity with histamine release magnitude correlating to cationic charge density (Hook et al., 1990). ELISA and flow cytometry quantify antibody titers demonstrating cross-reactivity with endogenous defensins; NetMHC algorithms identify T-cell epitopes with IC50 < 500 nM as putative immunogenic risk thresholds (Trier and Houen, 2023). Humanized sequence components mitigate this feature, yet longitudinal immunogenicity data across genetically diverse cohorts remain lacking (Schubert et al., 2017). Guryanova and Ovchinnikova (2022) validated foreign epitope-triggered IgE synthesis causes anaphylaxis-like reactions proportional to cationic charge density.

Aggregation compromises aqueous solubility through hydrophobic interaction-driven self-assembly into β-sheet fibrillar structures at elevated concentrations (Nomoto et al., 2021). Tryptophan-rich peptides undergo visible precipitation exceeding 1 mg/mL, lowering effective molarity and limiting bioavailability. Dynamic light scattering quantifies aggregate radii (10–100 nm) with polydispersity indices inversely correlating to membrane incorporation rates (Choi et al., 2018). Yang et al. (2024) demonstrated hydrophobic interactions drive β-sheet fibril formation compromising solubility; DLS revealed 10–100 nm aggregates whose polydispersity inversely correlated with microbial membrane incorporation. MD simulations identify π-π stacking among aromatic side chains as critical thermodynamic determinants of aggregation propensity.

Manufacturing costs reflect labor-intensive solid-phase peptide synthesis (SPPS) via Fmoc protection, where coupling yields drop below 50% for sequences exceeding 20 residues due to accumulation of deletion sequences (Sun et al., 2018). Consequently, extensive HPLC purification is required to achieve ≥95% purity, driving clinical-grade peptide costs above $100/g. Recombinant production in *E. coli* offers higher theoretical yields but introduces endotoxin removal costs that inflate prices 10–20 × versus small-molecule antibiotics. Su et al. (2025) confirmed SPPS yields drop below 50% beyond 20 residues driving costs above $100/g; recombinant *E. coli* production requires costly endotoxin removal inflating prices 10–20 × conventional antibiotics. Oxidative degradation involving methionine attack and asparagine deamidation exacerbated by moisture limits shelf life to < 6 months at ambient temperature per ICH recommendations. Arrhenius formalism yields activation energies of 80–100 kJ/mol for disulfide-containing AMPs (Evans et al., 2020). Lyophilization with trehalose cryoprotectants broadens stability yet scalable formulations retaining integrity under tropical conditions remain elusive.

PEGylation coupled with cyclization substantially augments stability. Polyethylene glycol attachment increases hydrodynamic radius diminishing renal filtration and protease access; PEGylated AMP conjugates exhibit 5–10 × half-life prolongation (Vernen et al., 2019). Head-to-tail or disulfide cyclization imparts conformational rigidity conferring exopeptidase resistance. Preclinical studies confirm cyclized PEGylated variants sustain antimicrobial concentrations well beyond initial dosing intervals (Chaudhary et al., 2016).

### 6.2 Toxicity and selectivity concerns

Hemolytic activity against red blood cells (RBCs) stems from non-selective lipid bilayer disruption quantified by HC50 (concentration causing 50% hemolysis) (Zhang et al., 2024). Cationic peptides like melittin partition into zwitterionic phosphatidylcholine-rich RBC membranes creating pores releasing hemoglobin (Luo et al., 2023). Contemporary modeling links hemolytic profiles to amphipathicity index (μH ≥ 0.4) using quartz crystal microbalance recording association rate constants (Ka) of 10^5^–10^7^ M^−1^. Structure–activity optimization truncating hydrophobic tails reduces HC50/MIC ratios < 10 enhancing microbial membrane selectivity (Elliott et al., 2020). Rathore et al. (2025) confirmed non-selective RBC membrane partitioning causes hemolysis quantifiable by HC50; hydrophobic tail truncation successfully reduced HC50/MIC ratios below 10 improving selectivity.

Off-target commensal microbiota toxicity disrupts mucosal homeostasis, promoting dysbiosis through broad-spectrum action that deactivates beneficial strains including *Lactobacillus*. In specific murine co-culture models using cationic AMP cocktails, broad-spectrum AMP administration was associated with 20–40% reductions in gut microbiota alpha-diversity via 16S rRNA surveys (Amoroso et al., 2020), accompanied by increased *Clostridium difficile* abundance. These effects are highly context-dependent and vary significantly by peptide class, dose, and host physiology; extrapolation to human clinical settings requires caution. From a One Health perspective, such dysbiosis extends beyond individual patients: disrupted gut ecosystems may potentially shed resistant opportunists into wastewater and soil, creating environmental reservoirs that could feed back into human and animal exposure cycles. Probiotic co-administration partially counterbalances these changes, yet long-term ecological consequences necessitate extended longitudinal investigation (Säll et al., 2023).

Nephrotoxicity originates from cationic AMP retention in proximal tubular cells binding anionic microvillous membranes provoking degeneration tracked by kidney injury molecule-1 (KIM-1) biomarker (Campbell et al., 2023). Doses > 10 mg/kg induce distinct tubular vacuolar degeneration reflected in rising histological injury scores. Toxicokinetic modeling accounting for megalin-mediated glomerular endocytosis enables NOAEL determination through established exposure parameters. Neutral inert polymer co-administration mitigates renal retention facilitating urinary AMP clearance (Kimura et al., 2020).

Bacterial resistance emerges through membrane remodeling modifying lipid A moieties neutralizing cationic attraction. Serial passage in AMP-rich media reveals stepwise MIC increases of 4–32 × over 10–20 generations (Guo et al., 2021). Whole-genome sequencing exposes *phoP/Q* two-component regulatory system mutations driving multidrug efflux pump overproduction; mutants incur 10–20% growth rate fitness penalties. Combination therapy with antioxidants or cell-wall-active agents imposes simultaneous divergent selective pressures mitigating resistance emergence. Zhang et al. (2025) confirmed lipid A modification neutralizes cationic attraction; serial passage showed 4–32 × MIC increases over 10–20 generations with *phoP/Q* mutations upregulating efflux pumps.

Host genetic variability drives clinical response divergence. Matrix metalloproteinase SNPs modify peptide half-life producing divergent outcomes. GWAS catalogues ABC efflux transporter polymorphisms correlating with AUC changes accounting for 20–30% therapeutic outcome variability. Therapeutic drug monitoring tailors dosing to individual patient profiles enabling precise clinical management. Predictive toxicology models relying on simplified immortalized cell line assays underestimate systemic toxicity with false-negative rates >30% due to deficient metabolic capacity in standard 2D configurations (Raies and Bajic, 2016). iPSC-derived kidney and liver organoids demonstrate physiologically relevant epithelial barriers and cell–cell interactions integrable into high-throughput toxicity assays; organoid-generated LD50 estimates converge with *in vivo* results in 80–90% of cases, providing a robust hazard prediction foundation (Czerniecki et al., 2018). Furthermore, advanced methodologies, such as 3D refractive index tomography, are increasingly utilized to visualize and quantify the progressive hemolytic activity and morphological changes in red blood cells induced by cationic peptides (Hur et al., 2017).

### 6.3 Regulatory and economic hurdles

FDA/EMA regulatory frameworks mandate exhaustive safety/performance delineation. IND dossiers pair non-clinical data with comprehensive Chemistry, Manufacturing, and Control (CMC) documentation specifying ≥ 95% purity via HPLC. First-in-class entities employ 505(b)(1) pathways necessitating successive Phase I-III trials. Quality by Design (QbD) tenets invoke detailed impurity hazard quantifications (e.g., truncated isoforms) and stipulate bioequivalence benchmarks for generics predicated upon 80–125% acceptance intervals for AUC/Cmax ratios. Orphan drug designation compresses development timelines for ultra-rare disease therapeutics (Grangeia et al., 2019). Zane et al. (2020) emphasized CMC documentation requires ≥ 95% HPLC-purity alongside detailed non-clinical safety data.

Patent challenges for naturally sourced AMPs pivot on novelty hurdles extending beyond sequence delineation. APD3 and similar repositories undercut composition-of-matter claims. Sophisticated protections extend to formulation/structural/functional modifications though enablement statutes oblige *in vivo* corroboration. Obviousness determinations under 35 U. S. C. §103 yield < 50% prevailing probability for AMP claims (Kosikowska and Lesner, 2016). Total peptide therapeutic expenditure routinely exceeds $1 billion benchmarked to small molecules (Lombardi et al., 2025). Multi-step chiral-resolved synthesis and multifaceted clinical programs drive Phase III trial budgets > $200 million. NPV simulations incorporating 90% pre-clinical attrition probabilities yield viable ROI projections only for blockbuster candidates. Biosimilar products modestly attenuate market barriers yet necessitate stringent comparability documentation affirming structural/functional interchangeability. Luo et al. (2023) highlighted poor oral bioavailability, enzymatic degradation, and cytotoxicity create significant translation hurdles addressable through nanoparticle/self-assembled carrier delivery systems.

Generic antibiotic competition impedes AMP uptake. Generic formulations price at marginal cost while AMPs command 10–50 × premiums. Porter’s five forces framework underscores payer bargaining power demanding cost-effectiveness < $50,000/QALY (Ji et al., 2024). Optimal penetration hinges on communicating proprietary resistance profiles promising MDR pathogen advantages. Fitch et al. (2021) confirmed generic antibiotics priced at marginal cost severely impede AMP adoption given 10–50 × premiums; payer < $50,000/QALY demands necessitate superior MDR efficacy demonstration justifying premium pricing.

Animal model ethical dilemmas center on welfare considerations tied to regulatory standards. Foundational 3Rs (Refinement, Reduction, Replacement) replicate key phenomenological elements in zebrafish larva assays decreasing mammalian model reliance (Gorzalczany and Basso, 2021). Murine-human pharmacodynamic discordance rates exceed 40% prompting *in silico* ADMET computational pipeline refinement prior to *in vivo* experimentation (Ferreira and Andricopulo, 2019).

Global access discrepancies stem from prohibitive pricing and fragile LMIC health infrastructures. Paucity of GMP facilities aggravates inequitable distribution; WHO access framework indicates AMPs reach < 10% sub-Saharan African formularies. Current databases lack comprehensive post-market data for >30 AMP candidates with <5-year follow-up, creating uncertainty regarding chronic immunogenicity or delayed resistance emergence (Oliveira et al., 2024). Standardized registry development is therefore advocated to monitor adverse events and real-world effectiveness beyond controlled trial environments (Deshpande et al., 2020; Mazurkiewicz-Pisarek et al., 2023; Gan et al., 2021).

## 7 Future perspectives and conclusions

The future of AMP research depends on integrating biotechnological innovation with systemic reforms that capitalize on untapped biological resources while addressing AMR through scalable, ethical, and economically viable frameworks extending beyond terrestrial applications (Gholizadeh et al., 2020). This section outlines the emerging technologies, policy innovations, and strategic conclusions required to translate AMPs from laboratory discoveries into reliable clinical therapeutics within a One Health context.

### 7.1 Advanced genetic, computational, and AI technologies

CRISPR-Cas systems enable endogenous AMP production in living hosts by precisely inserting AMP-encoding open reading frames into chromosomal loci of probiotic microbes or mammalian immune cells (Saha et al., 2024). Base editors and prime editors now permit seamless, footprint-free integration of AMP genes under tightly controlled inducible promoters like Tet-On, facilitating pathogen-responsive peptide expression (Kadkhoda et al., 2024). Multiplexed CRISPR arrays simultaneously knock out pathogen resistance determinants and activate dormant AMP loci; in murine infection models, such bioprotective circuits achieve >90% bacterial burden reduction *in vivo* against WHO priority pathogens. Phage-assisted continuous evolution iterates engineered Cas nucleases for enhanced specificity and minimized off-target editing, confirming that multiplexed CRISPR strategies can achieve profound *in vivo* bacterial reductions while evolved Cas systems minimize off-target effects (Yokoo et al., 2021; Devi et al., 2023).

mRNA-mediated delivery achieves transient AMP expression via lipid nanoparticles (LNPs) containing pseudouridine-modified transcripts that evade immune detection and extend half-life. Self-amplifying mRNA (saRNA) systems incorporating RNA-dependent RNA polymerase yield 10–100 × protein amplification, attaining peak concentrations within 24 h and clearing by 72 h to minimize prolonged subtherapeutic exposure (Blakney et al., 2020; Zalatan et al., 2015). Hybrid constructs fusing mRNA-encoded AMPs with CRISPR RNA scaffolds promise multifunctional gene editing and peptide delivery capabilities refined through codon optimization and advanced 5′ cap analogues enhancing translational efficiency (Pirtskhalava et al., 2020).

Multi-omics integration merges genomics, proteomics, and metabolomics to anticipate peptide functionality. Graph neural networks encode relational mappings spanning omics layers, enabling structure–activity prediction systems that achieve >85% accuracy for uncharacterized peptides (Fang Y. et al., 2024). Single-cell transcriptomics resolves host-pathogen interplay, paving the way for *de novo* AMP design calibrating antimicrobial spectra via variational autoencoders trained on integrative datasets (Cao and Gao, 2022). Xu et al. (2023) emphasized that stability enhancement strategies-including unnatural amino acid substitution, cyclization, protease inhibitor conjugation, and nanotechnology constraining conformational dynamics-effectively mitigate protease degradation and high-salt efficacy loss.

Furthermore, AI-optimized clinical trials accelerate AMP translation via predictive algorithms stratifying patients by genomic signatures, enhancing cohort enrollment efficiency (Vora et al., 2023). Bayesian adaptive designs modify endpoints in real-time; recent Phase II studies targeting Gram-negative pathogens have contracted timelines by 30–50% and enhanced statistical power through synthetic control arms derived from historical matched cohorts (Zhao Y. et al., 2022). Federated-learning architectures enable multinational collaborators to harmonize datasets without compromising confidentiality, fine-tuning dosing for genetic diversity (Duan et al., 2021; Cardoso et al., 2020).

### 7.2 Innovative manufacturing, delivery, and data security

Sustainable AMP production relies on synthetic biology platforms mitigating supply-chain risks. Recombinant expression in engineered *E. coli* and *Saccharomyces cerevisiae* with codon optimization maximizes translation efficiency; chassis modifications reprogramming metabolic pathways and introducing oligomeric fusion tags deliver titres exceeding grams/litre while decreasing environmental impact (Brooks and Alper, 2021). Cell-free expression systems integrated into biorefineries consume minimal reagents and energy, supporting rapid prototyping of optimized candidates (Silverman et al., 2019; Fu et al., 2025).

Additive manufacturing is revolutionizing AMP formulation via on-demand production of personalized dosage forms. Extrusion-based bioprinting employs AMP-encapsulated hydrogels as bioinks to assemble tablets and transdermal patches with bespoke release profiles (Nordström and Malmsten, 2017). Stereolithography integrates stimuli-responsive polymers inducing pH-triggered AMP release in prototype orthopedic implants with print resolutions down to 50 μm (Carratalá et al., 2020). While still in early preclinical stages, 4D printing utilizes shape-memory materials dynamically reconfiguring in wound environments, achieving >95% encapsulation efficiency in prototypes (Acosta et al., 2020; Zhao W. et al., 2022).

Wearable biosensing systems combine microfluidics with reservoir-fluidics for responsive AMP dosing driven by physiological readouts. Conformable adhesive films release peptide boluses in response to acidic wound microenvironments (Zhou et al., 2024). Microelectromechanical pumps and electrochemical modulators fine-tune flux, while aptamer-based biosensors yield pathogen-specific activation, achieving 1–10 μg/cm^2^ release in <3 s. Bluetooth-enabled nitric oxide-dispensing devices sustain therapeutic concentrations for 24 h with diminished cytotoxicity via closed-loop pH/voltage control (Nordström and Malmsten, 2017).

Proposed future applications include space biomedical applications where AMPs could potentially leverage against altered microbial virulence in microgravity. AMP-modified spacecraft surfaces obstruct biofilm establishment, exploiting polycationic amphiphilicity against Gram-negative organisms acquiring tolerance in low-g conditions (Xu et al., 2021). Custom-length amphiphilic peptides deployed in ISS potable-water filtration achieve 80–90% bioburden reduction against microgravity-evolved *Pseudomonas* strains. NASA roadmaps project AMP incorporation into regenerative life-support bioreactors using bacteriophage-AMP co-dosing to suppress resistance over prolonged exploration missions.

To secure the data driving these innovations, emerging blockchain technology frameworks could potentially secure AMP databases through decentralized immutable ledgers using hashing and smart contracts, ensuring data integrity and stratified access control. Zero-knowledge proofs enable privacy-preserving similarity searches without disclosing proprietary sequences, leveraged by globally distributed consortia for patent sharing (Saha et al., 2020). Hybrid blockchain-AI systems are projected to integrate multi-omics data streams, enabling real-time updates and consensus validation across transnational consortia, potentially reducing data silos by ~70% in AMR research networks (Marczak et al., 2025; Mohammadipanah and Sajedi, 2021; Belay et al., 2024).

### 7.3 Global policy, surveillance, and economic frameworks

Collaborative international initiatives refine AMR surveillance through federated databases underpinning strategic AMP administration. The WHO-endorsed Global Antimicrobial Surveillance System assesses clinical and epidemiological AMP impact (Kim et al., 2024). One Health frameworks synthesize veterinary and environmental resistance data via transatlantic working groups using genomic surveillance to monitor mutations in near-real time; machine-learning algorithms forecast resistance trajectories pre-emptively, directing AMP development and deployment (Djordjevic et al., 2023; Waddington et al., 2022).

Health economists are advancing equitable AMP pricing through value-based reimbursement models incorporating explicit resistance reduction modeling. Subscription-style payment frameworks disassociate revenue from sales volume; pull incentives may underwrite $1 billion developmental expenditures per qualifying candidate meeting clinical and resistance-mitigating benchmarks (Cama et al., 2021). Expanded health-technology assessments in low-resource settings validate public subsidies by clarifying quality-adjusted-life-year (QALY) gains (Dobrova et al., 2023).

Finally, standardized CLSI-compliant MIC testing using cation-adjusted Mueller-Hinton broth is essential for reconciling hemolysis and efficacy discrepancies. Advanced frameworks integrate biofilm models and pharmacokinetic-pharmacodynamic (PK-PD) correlates to enhance reproducibility (Ersoy et al., 2017). Policy landscapes promote AMP development via the GAIN Act extended exclusivity and delinked pricing models delivering $2–4 billion annually, underscored in recent UN resolutions designating AMPs as pivotal in global AMR containment (Yu et al., 2025).

## 8 Conclusion

Accumulated evidence positions AMPs as fast-acting, mechanistically diverse agents whose therapeutic potential requires careful design and disciplined translation. Their membrane disruption and immunomodulation capabilities distinguish them from traditional antibiotics, explaining their strongest clinical signals in high-local-concentration contexts: skin/soft tissue infections, wound care, mucosal surfaces, device coatings, and combination therapies overcoming permeability barriers. However, while these multifaceted mechanisms reduce the likelihood of rapid resistance emergence compared to single-target antibiotics, bacterial adaptation via lipid A modification, efflux pump upregulation, and protease secretion remains a documented challenge requiring active stewardship. Physicochemical features conferring antibacterial power also pose risks: protease susceptibility shortens systemic half-life; hydrophobic surfaces cause hemolysis and aggregation; renal handling concentrates toxicity; and manufacturing yields and purification increase costs. These challenges favor targeted, indication-specific strategies incorporating sequence, formulation, and delivery optimization from inception.

Three primary implications emerge from this review. First, encode selectivity through precise amphipathic moment, helicity, and oligomerization control validated by biophysics and organoid assays, maintaining potency while expanding therapeutic windows. Second, enhance combination strategies pairing membrane disruption with intracellular targets (e.g., lipid II/ribosome engagement) and conventional antibiotics to impose divergent selective pressures that mitigate-but do not eliminate-resistance development, boosting biofilm efficacy assuming standardized, biofilm-aware, CLSI-aligned susceptibility testing. Third, incorporate delivery as a core mechanism: cyclic/retro-inverso backbones resist proteases; depot/triggered-release systems control exposure; and transient *in situ* expression via mRNA or engineered microbes achieves high local concentrations without systemic peaks.

Persistent challenges-including planktonic MIC overreliance, limited toxicity models, fragile late-stage economics, and the inevitability of adaptive resistance under sustained selective pressure-require clearer design rules, shared data standards, adaptive trial designs, and revenue-use decoupling incentives. Aligning scientific and policy levers will enable AMPs to evolve from elegant biophysical innovations into reliable, context-aware therapeutics that supplement antibiotics, safeguard critical niches, and slow the progression of AMR.
