# Correction: Antimicrobial peptides in the era of resistance: integrating AI-driven design, host-microbe interactions, and nanotechnological delivery

**DOI:** 10.3389/fmicb.2026.2021107

**Published:** 2026-10-21

**Authors:** Frontiers Production Office

**Affiliations:** Frontiers Media SA, Lausanne, Switzerland

**Keywords:** antimicrobial peptides, antimicrobial resistance, artificial intelligence, CRISPR-Cas, drug delivery, host-microbe interaction, immunomodulation, nanotechnology

Author Wasim Ullah Khan was omitted as an author in the published article.

The correct author list reads:

Wasim Ullah Khan^1^, Zhu Yu Hua^2^, Xu Xia^2*†^, Khan Qaisar^3†^, Chen Zhong^1, 4, 5^, Khan Muhammad Shehzad^6, 7^ and Rashid Khan^8^

^1^School of Information Engineering, Yango University, Fuzhou, China

^2^School of Nursing, Shandong XieHe University, Jinan, Shandong, China

^3^School of Medicine, Yangtze University, Jingzhou, Hubei, China

^4^Institute of Industrial Internet Intelligent Control Technology and System in Universities of Fujian Province, Fuzhou, Fujian, China

^5^Institute of Intelligent Algorithms and Complex Systems, Yango University, Fuzhou, China

^6^Hong Kong Centre for Cerebro Cardiovascular Health Engineering, Hong Kong, Hong Kong SAR, China

^7^School of Medicine, City University of Hong Kong, Hong Kong, Hong Kong SAR, China

^8^School of Artificial Intelligence, Shenzhen Technology University, Shenzhen, China

The Author Contributions Statement has been corrected to read:

WK: Writing – original draft, Writing – review & editing, Methodology, Conceptualization, Formal analysis, Validation, Investigation, Resources, Visualization. XX: Conceptualization, Methodology, Formal analysis, Visualization, Investigation, Writing – review & editing, Validation. KQ: Conceptualization, Visualization, Writing – review & editing, Investigation, Formal analysis, Validation, Methodology. CZ: Formal analysis, Project administration, Writing – review & editing, Conceptualization.

KS: Methodology, Data curation, Formal analysis, Writing – review & editing, Software. RK: Formal analysis, Writing – review & editing, Visualization, Conceptualization, Data curation. ZH: Methodology, Formal analysis, Visualization, Investigation, Writing – review & editing, Validation.

The original version of this article has been updated.

